# Plant-Derived Extracellular Vesicles as a Delivery Platform for RNA-Based Vaccine: Feasibility Study of an Oral and Intranasal SARS-CoV-2 Vaccine

**DOI:** 10.3390/pharmaceutics15030974

**Published:** 2023-03-17

**Authors:** Margherita A. C. Pomatto, Chiara Gai, Federica Negro, Lucia Massari, Maria Chiara Deregibus, Cristina Grange, Francesco Giuseppe De Rosa, Giovanni Camussi

**Affiliations:** 1 EvoBiotech s.r.l., 10122 Turin, Italy; 2 Department of Medical Science, University of Turin, A.O.U. Città della Salute e della Scienza di Torino, 10126 Turin, Italy

**Keywords:** plant, exosome, extracellular vesicle, delivery, COVID-19, SARS, vaccine, RNA, oral, intranasal

## Abstract

Plant-derived extracellular vesicles (EVs) may represent a platform for the delivery of RNA-based vaccines, exploiting their natural membrane envelope to protect and deliver nucleic acids. Here, EVs extracted from orange (*Citrus sinensis*) juice (oEVs) were investigated as carriers for oral and intranasal SARS-CoV-2 mRNA vaccine. oEVs were efficiently loaded with different mRNA molecules (coding N, subunit 1 and full S proteins) and the mRNA was protected from degrading stress (including RNase and simulated gastric fluid), delivered to target cells and translated into protein. APC cells stimulated with oEVs loaded with mRNAs induced T lymphocyte activation in vitro. The immunization of mice with oEVs loaded with S1 mRNA via different routes of administration including intramuscular, oral and intranasal stimulated a humoral immune response with production of specific IgM and IgG blocking antibodies and a T cell immune response, as suggested by IFN-γ production by spleen lymphocytes stimulated with S peptide. Oral and intranasal administration also triggered the production of specific IgA, the mucosal barrier in the adaptive immune response. In conclusion, plant-derived EVs represent a useful platform for mRNA-based vaccines administered not only parentally but also orally and intranasally.

## 1. Introduction

Membrane vesicles secreted by cells have been described as a well-preserved evolutionary mechanism involved in communication among cells [1,2]. These vesicles originally described in eukaryotes, including mammalians, were subsequently found to be present in prokaryotes and in plants [3]. Virtually released by any cell type, secreted membrane vesicles are a heterogenous population with a complex structure that reproduces cell complexity at the nanoscale [1,2]. In fact, they are constituted by a lipid bilayer membrane containing several cell constituents that can be shared from the cell of origin with other cell types. This mechanism is the basis for a cell-to-cell mediated transfer of information [1,2,4]. Interestingly, the discovery of a horizontal transfer of vesicle-encapsulated nucleic acids [5,6,7,8,9,10] protected from enzyme degradation by the vesicle membrane opened up a new field of research that is now rapidly expanding. The term “extracellular vesicles” (EVs) [11] was proposed to include in this heterogenous population membrane vesicles released from multivesicular bodies, also named exosomes, and membrane vesicles originated by budding of plasma membrane, termed microvesicles or ectosomes. Independently of their origin, EVs share a common mechanism of action: the transfer of regulatory transcripts that target genes involved in defined pathways in recipient cells [5,6,7,8,9].

Of interest, the lipid bilayer membrane of EVs guarantees protection of nucleic acid from enzyme degradation, thus preserving the EV cargo [12]. Different reports showed the possibility to engineer EVs with an exogenous nucleic acid using the green fluorescent protein (GFP) mRNA that, once transferred to target cells, was translated into protein [6,7]. Several subsequent studies focused on the exploitation of EVs for drug delivery, with a particular interest in nucleic acids, showing that encapsulated mRNA, non-coding RNA, or DNA can be shared among cells [13]. In addition, strategies to load vesicles with selected membrane proteins have been developed. For instance, a fusion protein interacting with the Endosomal Sorting Complexes Required for Transport (ESCRT) cellular machinery was used to sort a chimeric protein into EVs [14]. The ability of EVs to transfer proteins or nucleic acids to the antigen-presenting cells (APCs) makes them a potential candidate as vaccine carriers [15]. Cells have been engineered to express proteins of SARS-CoV-2 in the EVs to develop a vaccine that induces long-lasting cellular and humoral responses [16]. Bacteria EVs decorated with Spike receptor-binding domain derived from mammalian cell culture were also used to generate anti-viral protein antibodies after intranasal administration [17]. Moreover, a potent SARS-CoV-2 CD8+ T immune response was obtained by generating engineered EVs released by muscle cells [18].

EVs from human cells can be engineered for encapsulating therapeutic agents due to the ability to transfer a great number of biomolecules [19]. However, scalable production of EVs from human cells in GMP conditions is difficult, time-consuming and highly expensive. With respect to human cell-derived EVs, plant-derived EVs may represent a good option for drug delivery because they are non-toxic, are an extractive product that can be produced on a large scale and can be directly manipulated and modified with a wide range of agents [20,21,22,23,24].

In the present study, we evaluated the possible use of edible plant-derived EVs as a delivery platform for mRNA-based vaccines. In fact, due to their resistance, plant-derived EVs represent an ideal natural source for the delivery of drugs such as small interfering RNAs (siRNAs), microRNAs (miRNAs) or poorly soluble natural compounds (e.g., curcumin) [24]. Plant EVs have the advantage, with respect to synthetic nanoparticles, of a high cellular internalization rate, good gastrointestinal stability and low intrinsic immunogenicity [25]. The aim of the present study was to evaluate whether plant-derived EVs were suitable for the delivery of SARS-CoV-2 mRNA as a vaccine via oral and intranasal administration routes. We tested three different SARS-CoV-2 mRNAs coding for full-length surface glycoprotein (FS) (commonly known as S protein), Spike-RBD subunit of S protein (S1) and nucleocapsid phosphoprotein (N). For this purpose, we directly engineered EVs extracted from an easily obtainable liquid source, orange (*Citrus sinensis*) juice, (oEVs) with a proprietary technique and evaluated in vitro the efficiency of mRNA molecules’ loading and their protection from degrading stress (RNase enzymes and simulated gastric fluid), the EV delivery of mRNA into target cells, the mRNA translation into protein and the subsequent activation of lymphocyte response. We investigated in vivo the effective production of serum antibodies versus the specific S protein of SARS-CoV-2, the specific immune cell activation and the effect of multiple routes of administration including intramuscular, oral and intranasal routes.

## 2. Materials and Methods

### 2.1. oEV Isolation and Loading

oEVs were isolated from freshly squeezed orange juice from *Citrus sinensis*, type Tarocco, purchased from a local certified organic producer (Arancebio srl, Francofonte, SR, Italy) in January 2021 and throughout 2022. The orange juice was filtered with a strainer and centrifuged at 4000× *g* for 30 min. The supernatant was ultracentrifuged at 10,000× *g* for 1 h at +4 °C (Optima L-90K ultracentrifuge, rotor 45 Ti, polycarbonate tubes, Beckman Coulter, Milan, Italy). The supernatant was then filtered to reduce the presence of fibers and re-ultracentrifuged at 100,000× *g* for 2 h at +4 °C (Optima L-90K ultracentrifuge, rotor 45 Ti, polycarbonate tubes, Beckman Coulter, Milan, Italy). The pellet was re-suspended in saline solution (NaCl 0.9%, B. Braun, Milan, Italy) added with 1% DMSO (Sigma-Aldrich, Merck, Darmstadt, Germany), filtered with 0.22 filters (Millex, Millipore, Merck, Darmstadt, Germany) for sterilization and stored at −80 °C for further experiments.

mRNA sequences were designed as described in Table 1 and purchased from RiboPro (Oss, The Netherlands). No codon optimization or other mRNA modifications were applied to mRNA sequences. The mRNAs were completed with a 5’UTR designed for high expression, a poly-A tail and a Cap1 with methylation of the first nucleotide to produce a Cap1 structure. After mRNA synthesis, dsRNA was removed to avoid innate immune-mediated translational repression. mRNAs were loaded into oEVs with a proprietary technique described in the patent application WO/2022/152771A1 using cation-based interaction combined with controlled osmotic shock.

### 2.2. oEV Characterization

oEVs were analyzed through nanoparticle tracking analysis (NTA) using the NanoSight NS300 system (Malvern Panalytical, Malvern, UK), equipped with NTA 3.4 analytic software. The instrument uses a laser source to inspect the sample and analyzes the Brownian movements of detected particles. The analytic software uses the Stokes–Einstein equation for converting this information into size and concentration parameters. For each sample, oEVs were diluted in a range of 1:200–1:2000 in 1 mL of saline solution (NaCl 0.9%, B. Braun, Milan, Italy) previously filtered with 0.1 µm membranes (Millex, Millipore, Merck, Darmstadt, Germany). Three videos of 30 s duration were recorded and camera levels were set to 15 for all the acquisitions. Settings for NTA post-acquisition were optimized and maintained constant among all samples, and each video was analyzed to measure the mean size and concentration of oEVs.

The morphology and integrity of oEVs were analyzed by transmission electron microscopy (TEM) as previously described [26]. Briefly, oEVs were left to adhere on 200 mesh nickel formvar carbon-coated grids (Electron Microscopy Science, Hatfield, PA, USA) for 20 min. Then, grids were incubated with 2.5% glutaraldehyde plus 2% sucrose. After washing in distilled water, samples were negatively stained with Nano-W and NanoVan (Nanoprobes, Yaphank, NY, USA) and analyzed using a Jeol JEM 1400 Flash electron microscope (Jeol, Tokyo, Japan).

### 2.3. RNA Extraction and qRT-PCR

Total RNA was extracted from cells and oEVs using a miRNeasy mini kit (Qiagen, Hilden, Germany) following the manufacturer’s instructions. RNA was eluted in nuclease-free water (Ambion, Thermo Fisher Scientific, Waltham, MA, USA) and the RNA concentration was assessed by measuring the absorbance at 260 nm with a spectrophotometer (mySPEC, VWR, Radnor, PA, USA). Samples were stored at −80 °C until analysis.

RNA was retro-transcribed to cDNA using a High-Capacity cDNA Reverse Transcription Kit (Thermo Fisher Scientific, Waltham, MA, USA) following the manufacturer’s instructions (100 nanograms for cell analysis and 10 µL for oEV analysis). Syn-cel-miR-39 (Qiagen, Hilden, Germany) was added to oEV samples as spike-in during the retro-transcription procedure. For qRT-PCR, each sample was run in triplicate; the primers (Eurofins Genomics, Milan, Italy) are listed in Table S1. For syn-cel-miR-39, the universal primer (Qiagen, Hilden, Germany) was used as the reverse primer. Human ACTB was used as the endogenous control for mRNA incorporation into target cells, whereas cel-mir-39 was used as the endogenous control for mRNA loading into oEVs. cDNA (5 nanograms for cell analysis and 2.5 µL for oEV analysis) was combined with SYBR GREEN PCR Master Mix (Thermo Fisher Scientific, Waltham, MA, USA) as described by the manufacturer’s protocol. The Real-Time Thermal Cycler Quant Studio 12k and ExpressionSuite Software 1.0.3 (Thermo Fisher Scientific, Waltham, MA, USA) were used to calculate relative quantification (RQ) values via the 2^−ΔΔCt^ method.

For the generation of standard curves for absolute quantification of mRNAs, each mRNA (S1, FS and N) was spectrophotometrically quantified and 200 ng were reverse transcribed using the High-Capacity cDNA Reverse Transcription Kit (Thermo Fisher Scientific, Waltham, MA, USA). cDNA was serially diluted 1:5 from a starting point of 10 ng to have 11 dilutions. Serial dilutions were run in five replicates using Relative Standard Curve on a 96-well QuantStudio 12K Flex Real-Time PCR system, as described by the manufacturer’s protocol (Thermo Fisher Scientific Scientific, Waltham, MA, USA). The calibration curve was used to convert the Ct values of each sample into the corresponding amount of mRNA. The percentage of engineering yield was calculated as follows: (final amount mRNA/oEV)/(starting amount mRNA/oEV). EV loading was repeated in three independent experiments for each mRNA used.

### 2.4. PCR and Electrophoresis

cDNA was amplified with iProofTM High-Fidelity DNA Polymerase (Biorad, Hercules, CA, USA) following the manufacturer’s instructions. The PCR reaction mix was composed of 4 ng cDNA, 500 nM primer (Eurofins Genomics, Milan, Italy), 0.5 µL iProof DNA Polymerase (Biorad, Hercules, CA, USA), 10 µL 5x iProof HF buffer, 1 µL dNTPs, 0.5 µL MgCl2, and nuclease-free water (Thermo Fisher Scientific, Waltham, MA, USA) to reach the reaction volume of 50 µL. The CTR DNA template was amplified with 1.3 kB primers (both provided by the kit) as the internal reaction control. The PCR was performed with VERITI Thermal Cycler (Thermo Fisher Scientific, Waltham, MA, USA), with 30 amplification cycles run. For gel electrophoresis, 15 µL of PCR products was mixed with 3 µL of 6X TriTrack DNA Loading Dye from a GeneRuler 100 bp Plus DNA Ladder Kit (Thermo Fisher Scientific, Waltham, MA, USA). Then, 15 µL of the mix was loaded on a 5% Mini-PROTEAN^®^ TBE Gel (Biorad, Hercules, CA, USA). The electrophoresis cell was filled with 1X TBE buffer (Biorad, Hercules, CA, USA) and the run was performed at 100 V for 45 min. Gels were soaked for 20 min in ethidium bromide (Biorad, Hercules, CA, USA) diluted to 0.5 µg/mL in 1X TBE buffer and subsequently washed in sterile water (B.Braun, Milan, Italy) for 20 min at RT. Images were acquired with a ChemiDoc System (Biorad, Hercules, CA, USA). Data were obtained from three independent experiments.

### 2.5. Cell Culture

Human macrophages (MV-4-11), human dermal microvascular endothelial cells (HMEC-1) and normal human dermal fibroblasts (NHDF) were obtained from ATCC (Manassas, VA, USA) and cultured following the manufacturer’s instructions with, respectively, Iscove’s modified Dulbecco’s medium (IMDM, ATCC, Manassas, VA, USA), MCDB131 medium (Gibco, Thermo Fisher Scientific, Waltham, MA, USA) and fibroblast growth basal medium (FBM, Lonza, Basel, Switzerland) supplemented with 10% fetal bovine serum (FBS, Life Technologies, Thermo Fisher Scientific, Waltham, MA, USA). Human primary peripheral blood mononuclear cells (PBMC) were isolated from the peripheral blood of healthy donors provided by the Centro Produzione e Validazione Emocomponenti of the A.O.U. Città della Salute e della Scienza di Torino with internal ethical approval. PBMC were isolated via a density gradient using Histopaque^®^-1077 (Sigma Aldrich, Merck, Darmstadt, Germany), washed with PBS (Thermo Fisher Scientific, Waltham, MA, USA) and plated at a concentration of 2 × 10^6^ cells/mL in a 12-well plate with RPMI 1640 (Euroclone, Milan, Italy) supplemented with 10% FBS (Life Technologies, Thermo Fisher Scientific, Waltham, MA, USA). All cells were incubated at 37 °C with 5% CO_2_.

The effect of oEVs on cell viability was evaluated with a MTT Cell Growth Assay Kit following the manufacturer’s instructions (Sigma-Aldrich, Merck, Darmstadt, Germany). Briefly, HMEC-1 was plated at a density of 5000 cells/well in 96-well plates (Euroclone, Milan, Italy) and treated with four increasing doses of oEVs (10,000, 50,000, 100,000 and 200,000 particles/cell) diluted in medium (DMEM low glucose with 5% EV-depleted FBS) (Euro-clone, Milan, Italy). The viability measurement was performed after 24 h of treatment, comparing cells cultured with medium, medium plus oEVs or medium plus 50% DMSO as the positive control (Sigma-Aldrich, Merck, Darmstadt, Germany). Data were obtained from three independent experiments.

### 2.6. mRNA Stress Resistance

To test the resistance to enzyme degradation of mRNAs loaded into oEVs, samples were treated with 0.4 µg/mL Ambion^TM^ RNase A (Invitrogen, Thermo Fisher Scientific, Waltham, MA, USA) and incubated at 37 °C for 30 min as previously described [27]. The reaction was stopped by adding Ambion™ RNase inhibitor (Invitrogen, Thermo Fisher Scientific, Waltham, MA, USA) according to the manufacturer’s instructions. Samples were washed by ultracentrifugation at 100,000× *g* for 2 h at +4 °C using a 10 mL polycarbonate tube (SW 90 Ti rotor, Beckman Coulter Optima L-90 K ultracentrifuge, Beckman Coulter, Milan, Italy) and were resuspended in saline buffer solution for analysis. The resistance to gastrointestinal degradation was investigated as previously described [28]. Briefly, a simulated gastric fluid (SGF) was prepared using 18.5% *w*/*v* HCl (pH 2.0), 24 mg/mL of bile extract, pepsin solution (80 mg/mL in 0.1 N of HC_l_, pH 2.0) and 4 mg/mL of pancreatin in 0.1 N of N_a_HCO_3_ (Sigma-Aldrich, Merck, Darmstadt, Germany). Next, 100 µL of each oEV sample was incubated with 1.34 µL of SGF at 37 °C for 60 min. Then, the pH value was adjusted to 6.4 with 1 N N_a_HCO_3_ to mimic the intestinal solution and incubated for 60 min. Free mRNA was used as a control and the percentage of resistance was defined as the percentage of mRNA remaining after stress treatments in comparison to starting material. Data for each mRNA were obtained from three independent experiments. For experiments with Triton X-100, samples were treated with 1% Triton X-100 (Biorad, Hercules, CA, USA) for 1 h at +4 °C before RNAse treatment and molecular analysis.

### 2.7. mRNA Incorporation into Target Cells

Human macrophages were plated 60,000 cells/well in 24-well plates (Sarstedt, Milan, Italy) and treated with different stimuli: medium (IMDM plus 10% FBS), or medium plus oEVs, oEV-S1, oEV-FS or oEV-N (1.2 × 10^10^ oEVs containing 1 µg mRNA/well). As a control, cells were co-incubated or transfected with a similar amount of each mRNA with Lipofectamine^®^ 2000 Reagent (Thermo Fisher Scientific, Waltham, MA, USA) following the manufacturer’s instructions. Next, 0.75 µL/well lipofectamine and 1.0 µg/well mRNA were diluted in DMEM low glucose (Euroclone, Milan, Italy), incubated for 5 min at room temperature and added to wells containing medium. After 24 h, cells were harvested and processed for molecular analysis. Data were obtained from three independent experiments.

### 2.8. Cytofluorometric Analysis of mRNA Translation into Protein

HMEC-1 was plated at 50,000 cells/well in 24-well plates (Sarstedt, Milan, Italy) and treated with different stimuli: medium (MCDB131), or medium plus oEVs, oEV-S1, oEV-FS or oEV-N (1.2 × 10^10^ oEVs and 1 µg mRNA/well). As the control, cells were transfected with a similar amount of each mRNA using Lipofectamine^®^ 2000 Reagent (Thermo Fisher Scientific, Waltham, MA, USA) following the manufacturer’s instructions. Briefly, 0.75 µL/well lipofectamine and 1.0 µg/well mRNA were diluted in DMEM low glucose (Euroclone, Milan, Italy), incubated for 5 min at room temperature and added to each well containing MCDB131 medium plus 10% FBS. Treatment with only lipofectamine reagent was used as the transfection control. After 24 h of treatment, cells were collected and treated using an Inside Perm Kit (Miltenyi Biotec, Bologna, Italy) for the intracellular staining, following the manufacturer’s instructions. Briefly, cells were treated with Fix Inside reagent for 20 min at room temperature, washed with PBS and mixed with anti-N-protein rabbit antibody (1:250, PA1-41098, Invitrogen, Thermo Fisher Scientific, Waltham, MA, USA) or anti-S1-protein mouse antibody (1:100, MA5-38033, Invitrogen, Thermo Fisher Scientific, Waltham, MA, USA) for 1 h at room temperature. After washing, samples were incubated with 5 µg/mL fluorescent secondary antibody anti-mouse (green, A32723, Invitrogen, Thermo Fisher Scientific, Waltham, MA, USA) or anti-rabbit (red, A32740, Invitrogen, Thermo Fisher Scientific, Waltham, MA, USA) for 1 h at room temperature. Finally, samples were washed and resuspended in saline solution for acquisition with CytoFLEX (Beckman Coulter, Milan, Italy) and CytExpert software (v 2.3.0.84, Beckman Coulter, Milan, Italy). Data were obtained from three independent experiments.

### 2.9. Confocal Microscopy Analysis of mRNA Translation into Protein

HMEC-1 were plated at 10,000 cells/well in 8-well chamber slides (Thermo Fisher Scientific, Waltham, MA, USA) and treated with different stimuli: medium (MCDB131), or medium plus oEVs, oEV-S1, oEV-FS or oEV-N using a particle dose of 1.2 × 10^10^ oEVs/well and 1 µg mRNA/well. As control, the cells were transfected with a similar amount of nude mRNA S1, FS, N. Transfection was performed with Lipofectamine^®^ 2000 Reagent (Thermo Fisher Scientific, Waltham, MA, USA) following the manufacturer’s instructions. Briefly, 0.16 µL/well Lipofectamine^®^ 2000 Reagent and 1.0 µg/well mRNA were diluted in DMEM low glucose (Euroclone, Milan, Italy), incubated for 5 min at room temperature and added to each well containing MCDB131 medium plus 10% FBS. Treatment with only Lipofectamine^®^ 2000 Reagent was used as the transfection control.

After 24 h of treatment, cells were washed with PBS, fixed with ice-cold methanol:acetone (1:1), incubated at −20 °C for 10 min and air dried. Then, wells were blocked with PBS with 1% bovine serum albumin (BSA, Sigma-Aldrich, Merck, Darmstadt, Germany) for 30 min at 37 °C, and after washing, were incubated with anti-N-protein rabbit antibody (1:250, PA1-41098, Invitrogen, Thermo Fisher Scientific, Waltham, MA, USA) or anti-S1-protein mouse antibody (1:100, MA5-38033, Invitrogen, Thermo Fisher Scientific, Waltham, MA, USA) for 1 h at RT. After three washes with PBS, samples were incubated with anti-mouse (1:1000, green, A32723, Invitrogen, Thermo Fisher Scientific, Waltham, MA, USA) or anti-rabbit (1:1000, red, A32740, Invitrogen, Thermo Fisher Scientific, Waltham, MA, USA) fluorescent antibodies for 1 h at room temperature, protected from light. After three washes with PBS, samples were incubated with DAPI at 300 nM (Thermo Fisher Scientific, Waltham, MA, USA) for 10 min at room temperature, protected from light. Then, samples were washed three times with PBS and air-dried. One drop of Fluormount Aqueous Mounting Media (Sigma Aldrich, Merck, Darmstadt, Germany) was added to each spot and the glass was covered with a coverslip. Images were acquired with the confocal microscope Axiovert 200M equipped with LSM5 Pascal and analyzed with LSM image browser (Zeiss, Oberkochen, Germany). Data were obtained from three independent experiments.

### 2.10. Cytofluorometric Analysis of oEV-GFP

GFP translation after oEV-mediated delivery was evaluated after 24 h of treatment of HMEC-1 was plated at 30,000 cells/well in 24-well plates (Sarstedt, Milan, Italy) with medium (MCDB131), or medium plus oEVs (1 × 10^10^ oEVs/well), oEVs loaded with GFP mRNA (oEV-GFP) (1 × 10^10^ oEVs and 0.83 µg mRNA/well) (OZ Biosciences, Marseilles, France), GFP mRNA or GFP mRNA transfected with lipofectamine. Transfection was performed with Lipofectamine^®^ 2000 Reagent (Thermo Fisher Scientific, Waltham, MA, USA) following the manufacturer’s instructions using 1.67 µg/well mRNA. After 24 h, cells were harvested and analyzed.

oEV uptake and GFP delivery were also investigated after RNase and SGF stress. For that purpose, oEVs were previously stained with 1:100 diluted PKH26 fluorescent dye (Sigma-Aldrich, Thermo Fisher Scientific, Waltham, MA, USA) for 30 min at 37 °C and washed by ultracentrifugation at 100,000× *g* for 2 h at +4 °C (SW 90 Ti rotor, Beckman Coulter Optima L-90 K ultracentrifuge, polycarbonate tubes, Beckman Coulter, Milan, Italy). Stained oEVs were loaded with GFP mRNA (oEV-GFP). oEVs (1.2 × 10^10^ oEVs/well), oEV-GFP (1.2 × 10^10^ oEVs and 0.5 µg mRNA/well) and 0.5 µg of free GFP mRNA were subjected to incubation with RNase and SGF and used to treat 20,000 cells/well NHDF in 24-well plates (Sarstedt, Milan, Italy). mRNA was transfected using Lipofectamine^®^ 2000 Reagent (Thermo Fisher Scientific, Waltham, MA, USA) following the manufacturer’s instructions.

Data for each mRNA were obtained from three independent experiments. Analysis was performed with CytoFLEX (Beckman Coulter, Milan, Italy) and CytExpert software (v 2.3.0.84, Beckman Coulter, Milan, Italy). Data were obtained from three independent experiments.

### 2.11. Lymphocytes’ Activation In Vitro

Human macrophages plated in 24 well-plates (Sarstedt, Milan, Italy) at a density of 20,000 cells/well were co-incubated with several stimuli: medium (RPMI with 10% FBS), medium plus oEV, oEV-S1, oEV-FS or oEV-N (1.2 × 10^10^ oEVs and 1 µg /well of mRNA), 2 µg/mL S protein and N protein of SARS-CoV-2 (RP-87706 and RP-87665, Thermo Fisher Scientific, Waltham, MA, USA) and activation beads at 10 µL/well (Dynabeads^®^ Human T-Activator CD3/CD28, Life Technologies, Thermo Fisher Scientific, Waltham, MA, USA). After 5 h, 200,000 PBMC were added to each well, and stimulation with treatments was repeated after 5 days. On day 10 of the experiment, cells were harvested and analyzed.

For cell proliferation analysis, PBMC were labeled with CellTrace™ CFSE dye (Thermo Fisher Scientific, Waltham, MA, USA) following the manufacturer’s instructions before incubation with macrophages. At the experiment endpoint, cells were collected and stained with CD4-APC antibody (1:50, 130-113-222, Miltenyi Biotec, Bologna, Italy) using an appropriate isotype.

For marker expression, cells were harvested and stained with CD4-FITC (1:50, MHCD0401, CALTAG Laboratories, Burlingame, CA, USA), CD25-APC-Vio^®^770, CD69-APC-Vio^®^770 or HLADR-APC-Vio^®^770 (1:100, 130-123-469, 130-112-616, 130-111-792, Miltenyi Biotec, Bologna, Italy) with appropriate isotypes.

Antibody staining was performed for 30 min and washed by centrifugation at 500× *g* for 10 min. Analysis was performed with CytoFLEX (Beckman Coulter, Milan, Italy) and CytExpert software (v 2.3.0.84, Beckman Coulter, Milan, Italy). Data were obtained from three independent experiments.

### 2.12. Mice Immunization and Sacrifice

The study was conducted according to the National Institute of Health Guidelines for the Care and Use of Laboratory Animals, and approved by the Ethics Committee of the University of Turin and the Italian Ministry of Health (authorization number 514/2021-PR, approved on 12 July 2021). Female BALB/cAnNCrl mice of 6–8 weeks old were purchased from the Department of Molecular Biotechnology and Health Sciences, University of Turin. Behavior and health status were observed daily and weight checked weekly. Immunization was performed by administering empty oEVs and mRNA-loaded oEVs (oEV-S1 or oEV-FS) via intramuscular (IM), oral and intranasal (IN) routes.

For IM and IN administration, animals received one immunization (equivalent to 7.2 × 10^11^ oEVs containing 60 µg mRNA in 100 µL for IM or 40 µL for IN route of saline solution for each dose) at day 0 and one booster immunization after 3 weeks. For intramuscular administration, treatments were injected into the same hind leg for both doses, whereas for intranasal administration, the dose was pipetted into mice’s nostrils. For oral administration, animals received one immunization (equivalent to 1.2 × 10^12^ oEVs containing 100 µg mRNA in 150 µL of a solution of 1% chitosan for each dose) at day 0 using gavage needle and three booster immunizations after 3 weeks on consecutive days.

Mice were euthanized after 2 weeks from the last booster immunization. The group named oEV-S1 received immunization with S1 mRNA (*n* = 6 for IM, *n* = 7 for IN and *n* = 9 for oral), whereas the group named oEV (*n* = 5 for IM, *n* = 7 for IN and *n* = 6 for oral) received an equivalent dose of unloaded oEV as a negative control. The group named oEV-FS received immunization with FS mRNA (*n* = 6 for IM, *n* = 3 for IN, and *n* = 2 for oral), whereas the group named oEV (*n* = 6 for IM, *n* = 4 for IN and *n* = 3 for oral) received an equivalent dose of unloaded oEV as a negative control.

Immediately after sacrifice, spleens were collected in HBSS (Thermo Scientific, Waltham, MA, USA) and processed for cell isolation. Blood was collected and centrifuged at 3.000× *g* for 20 min to obtain serum. Sera were stored at −20 °C until use. The bronchoalveolar lavage (BAL) fluid was collected from mice immunized with oEV (*n* = 2) or oEV-S1 (*n* = 3) and stored at −20 °C until use.

Muscle and intestine morphology was evaluated through formalin-fixed paraffin-embedded tissue staining. Paraffin tissue sections (5 µm thick) were routinely stained for microscopic evaluation with H&E (Merck, Darmstadt, Germany) and photographed with an Axioskop microscope (Zeiss, Oberkochen, Germany) equipped with a Canon DS126181 camera (Canon, Tokyo, Japan).

### 2.13. Antibody Titer Analysis

Specific IgG, IgM, and IgA antibodies against the S protein of SARS-CoV-2 virus in mice serum or BAL samples were detected through enzyme-linked immunosorbent assay (ELISA). For that purpose, Nunc Maxisorp ELISA plates (Thermo Fisher Scientific, Waltham, MA, USA) were coated overnight at 4 °C with 100 µL/well of 1 µg/mL SARS-CoV-2 S1 RBD recombinant protein (RP-87706, Thermo Fisher Scientific, Waltham, MA, USA) diluted in BupH carbonate–bicarbonate buffer (Thermo Scientific, Waltham, MA, USA). After three washes with PBS 1X, plates were blocked with 200 µL/well of PBS 1X–3% bovine serum albumin (BSA, Sigma Aldrich, Merck, Darmstadt, Germany) for 1 h at 37 °C. After five washes with PBS 1X—0.05% Tween20 (Sigma Aldrich, Merck, Darmstadt, Germany), plates were incubated with 100 µL/well of different serial dilutions of samples in duplicate (starting from dilution 1:100). Dilution buffer was also used as a blank: PBS 1X–0.05% Tween20–3% BSA for IgG, PBS 1X–3% BSA–5% FBS for IgM and PBS 1X–0.05% Tween20–3% BSA–5% FBS for IgA. Plates were incubated for 1 h at 37 °C for IgG, 2 h at room temperature for IgA or overnight at 4 °C for IgM. After five washes with PBS 1X–0.05% Tween20, 100 µL/well of secondary antibody was added: goat anti-mouse HRP IgG (1:10.000, 31430, Invitrogen, Thermo Fisher Scientific, Waltham, MA, USA), IgM (1:4.000, 62-6820, Invitrogen, Thermo Fisher Scientific, Waltham, MA, USA) or IgA (1:250, 8850450-88, Invitrogen, Thermo Fisher Scientific, Waltham, MA, USA). After 2 h incubation and five washes with PBS 1X–0.05% Tween20, 100 µL/well of Stabilized Chromogen (TMB, Life Technologies, Thermo Fisher Scientific, Waltham, MA, USA) was added and incubated for 15–30 min at room temperature. The reaction was stopped by adding 100 µL/well of ELISA Stop Solution (Invitrogen, Thermo Fisher Scientific, Waltham, MA, USA). The plates were read at 450 nm using a VICTOR^®^ Nivo™ Plate Reader (PerkinElmer, Milan, Italy) and VICTOR^®^ Nivo™ Control Software (v 4.0.7, PerkinElmer, Milan, Italy). End-point titers were calculated as the last dilution with an optical density ≥ 1.5 blank O.D. value or ≥ 2 blank O.D. value for IgA in BAL samples.

Specificity was evaluated using a competition assay as the percentage of reduction in O.D. value of serum samples (diluted 1:100 for IgM, 1:1000 for IgG and IgA), either control or pre-incubated with 30 µg/mL of SARS-CoV-2 S1 RBD recombinant protein for 1 h at 37 °C.

IgG sensibility was evaluated for the only mouse antibody anti-S protein available, IgG, using serial dilutions of SARS-CoV-2 Spike Protein (RBD) Monoclonal Antibody (MA5-38033, Invitrogen, Thermo Fisher Scientific, Waltham, MA, USA) ranging from 10 to 0.1 ng/mL following IgG protocol.

### 2.14. Neutralizing Antibody Analysis

A SARS-CoV-2 Neutralizing Antibody ELISA Kit (Invitrogen, Thermo Fisher Scientific, Waltham, MA, USA) was used to detect the level of neutralizing antibodies against SARS-CoV-2 in mice serum following the manufacturer’s instructions. Briefly, serum samples were diluted 1:50 with Assay Buffer 1X, and pre-coated plates were washed one time with Wash Buffer 1X. Next, 100 µL of positive control, Assay Buffer 1X (used as a negative control) and pre-diluted serum sample were added to the appropriate wells in duplicate and incubated for 30 min at room temperature. Plates were washed three times and 100 µL of Biotin Conjugate Solution 1X was added to each well. After incubation at room temperature for 30 min, plates were washed three times and 100 µL/well of streptavidin–HRP conjugate solution was added. Then, plates were incubated for 15 min at room temperature and washed three times, and 100 µL/well of substrate solution was added and left for 15 min at room temperature. The reaction was stopped by adding 100 µL/well of Stop Solution and plates were read on a VICTOR^®^ Nivo™ Plate Reader (PerkinElmer, Milan, Italy) using VICTOR^®^ Nivo™ Control Software (v 4.0.7, PerkinElmer, Milan, Italy) at 450 nm, using 620 nm as the reference wavelength. The percentage of neutralization was calculated using the following formula: 1 − (absorbance of unknown sample/absorbance of negative control) × 100.

### 2.15. Splenocytes’ Isolation

Mouse splenocytes were isolated from spleens freshly collected in HBSS (Thermo Fisher Scientific, Waltham, MA, USA). Spleens were disrupted and filtered through a 40 μm cell strainer (PluriSelect, Leipzig, Germany), diluted with PBS (Euroclone, Milan, Italy) and centrifuged at 500× *g* at 4 °C for 5 min. Each pellet was resuspended in 2 mL cold RBC lysis buffer 1X (Thermo Fisher Scientific, Waltham, MA, USA) and incubated on ice for 5 min to lyse red blood cells. The reaction was stopped with 10 mL ice-cold PBS and cells were pelleted at 400× *g* at 4 °C for 5 min and resuspended in RPMI 1640 supplemented with 10% FBS.

### 2.16. IFN-γ Detection with ELISPOT

Elispot plates (Merck Millipore, Darmstadt, Germany) were activated by adding 15 µL of 35% ethanol to each well, washed twice with PBS, coated with mouse IFN-γ capture antibody (part of Mouse IFN-γ ELISPOT Pair, 551881, BD Bioscience, Eysins, Switzerland) and left overnight at +4 °C. Plates were blocked using RPMI 1640 with 10% FBS for 2 h at room temperature. For each mouse, 3 × 10^6^ fresh splenocytes were plated in each well and re-stimulated with 2 µg/mL S peptide (SARS-CoV-2 S1 RBD recombinant protein, RP-87706, ThermoFisher Scientific, Waltham, MA, USA). For each condition, three technical replicates were performed. Plates were incubated for 44 h at 37 °C with 5% CO_2_. Cells were removed and plates were washed twice with deionized water and three times with washing buffer (ELISA wash buffer, Invitrogen, Thermo Fisher Scientific, Waltham, MA, USA). Next, they were incubated with mouse IFN-γ detection antibody for 2 h at room temperature. Then, plates were washed three times with washing buffer and incubated with streptavidin–HRP (557630, BD Bioscience, Eysins, Switzerland) for 1 h at room temperature. Following that, plates were washed four times with washing buffer and twice with PBS, then incubated with substrate solution (AEC substrate set, BD Bioscience, Eysins, Switzerland) for 15 min until spot development. The reaction was stopped by washing with sterile water, and plates were left to air-dry at room temperature in the dark until completely dry. Plate acquisition was performed using an ELISPOT plate reader with ImmunoSpot (S6 Macro M2, ImmunoSpot, Cleveland, OH, USA), and spot counting was performed automatically by ImmunoSpot Software version 7.0.21.0 (ImmunoSpot, Cleveland, OH, USA).

### 2.17. Cytokine ELISA Analysis

Mouse splenocytes were stimulated with 1 µg/mL of S peptide (SARS-CoV-2 S1 RBD recombinant protein, RP-87706, ThermoFisher Scientific, Waltham, MA, USA), and the supernatant was collected after 24 h, then centrifuged at 1400× *g* × 10 min to remove debris. Quantitative detection of mouse IFN-γ, IL-2, IL-10 and IL-4 levels was performed using a Mouse Th1/Th2 Uncoated ELISA Kit (88-7711-44, Invitrogen, Thermo Fisher Scientific, Waltham, MA, USA) according to the manufacturer’s instructions. Briefly, 96-well plates (Corning, Glendale, AZ, USA) were coated with 100 µL/well of capture antibody overnight at 4 °C. After washing, plates were blocked with 200 µL/well of ELISA/ELISPOT Diluent 1X for 1 h at room temperature and washed once with Wash Buffer. Mouse IFN-γ, IL-2, IL-10 and IL-4 standards were reconstituted, and two-fold serial dilutions were performed to produce a standard curve for a total of eight points. Next, 100 µL of samples or ELISA/ELISPOT Diluent as the blank were added to the well in duplicate and incubated for 2 h at room temperature. After washing, 100 µL/well of detection antibody was incubated for 1 h at room temperature. Then, 100 µL/well of Streptavidin-HRP was incubated for 30 min at room temperature and washed five times with Wash Buffer. Following that, 100 µL/well of TMB Solution 1X was incubated for 15 min at room temperature. The reaction was stopped by adding 100 µL/well of Stop Solution and plates were read on a VICTOR^®^ Nivo™ Plate Reader (PerkinElmer, Milan, Italy) using VICTOR^®^ Nivo™ Control Software version 4.0.7 (PerkinElmer, Milan, Italy) at 450 nm, subtracting the 570 nm reference wavelength. Standards curves based on standard O.D. values were used to calculate the cytokine amount (pg/mL) for each sample.

### 2.18. Cytofluorometric Analysis of Splenocyte-Derived Immune Cells

For ex vivo analysis, mouse splenocytes were stimulated with 1 µg/mL of S peptide (SARS-CoV-2 S1 RBD recombinant protein, RP-87706, ThermoFisher Scientific, Waltham, MA, USA). For marker expression, cells were harvested after 36 h and stained with Viobility Fixable Dye (Miltenyi, Bologna, Italy) and CD3ε-APC (1:50, 130-117-671), CD4-APC-Vio770 (1:50, 130-118-955), CD8-FITC (1:50, 130-122-720), CD69-PE (1:50, 30-115-460), CD3-FITC (1:50, 130-092-962), CD4-VioBlue (1:50, 130-123-208), CD25-PE (1:50, 130-120-696), CD8-FITC (1:50, 130-120-822), CD138-PE (1:50, 130-120-741), CD45R-FITC (1:50, 130-118-323) and appropriate isotypes for each fluorescence (Miltenyi, Bologna, Italy). After staining, cells were washed with PBS, centrifuged at 300× *g* for 10 min and resuspended in PBS for acquisition. For lymphocyte proliferation, cells were stained using a CellTrace™ CFSE cell proliferation kit (Invitrogen, Thermo Fisher Scientific, Waltham, MA, USA) before peptide stimulation in vitro following the manufacturer’s instructions. Briefly, 1 × 10^6^ splenocytes were incubated with 1 M CellTrace™ stock solution for 20 min at 37 °C and washed with RPMI 1640 supplemented with 10% FBS. Cells were plated at 1 × 10^6^ cells/mL and stimulated with 1 µg/mL of SARS-CoV-2 S1 RBD recombinant protein (RP-87706, Thermo Fisher Scientific, Waltham, MA, USA). After 5 days, cells were harvested and stained with CD4-APC-Vio770 antibody (1:50, 130-118-955, Miltenyi, Bologna, Italy) and an associated isotype. After staining, cells were washed with PBS, centrifuged at 300× *g* for 10 min and resuspended in PBS for acquisition with a CytoFLEX flow cytometer (Beckman Coulter, Milan, Italy) using CytExpert software (v2.3.0.84, Beckman Coulter, Milan, Italy).

### 2.19. Biodistribution

Male BALB/cAnNCrl mice were treated with fluorescently labeled oEVs using the IN and oral routes. oEVs were stained using 10 µM of Vybrant™ DiD Cell-Labeling Solution (Thermo Fisher Scientific, Waltham, MA, USA). The same amount of dye (DiD) used for oEV staining was administered as a control to set the background. Treatment was performed with 2 × 10^12^ oEVs for oral or 1.5 × 10^12^ oEVs for IN administration in one single dose (*n* = 5 for stained DiD oEVs, *n* = 3 for DiD). After 18 h, mice were euthanized and single organs were collected and imaged using an IVIS 200 small animal imaging system (PerkinElmer, Milan, Italy) as previously described [26]. Briefly, the excitation filter was set at 640 nm and the emission filter at 700 nm. The same illumination settings, such as binning factor (4), exposure time (2 s), f/stop (2) and field of views, were used for acquiring all images. The fluorescence signal was normalized to photons per second per centimeter squared per steradian (p/sec/cm^2^/sr). Images were acquired and analyzed using Living Image 4.0 software (PerkinElmer, Milan, Italy). The background average photon emission was subtracted from images to normalize the signal. The fluorescence (p/sec/cm^2^/sr) was quantified in the region of interest (ROI) drawn freehand. Data were expressed as average radiance ± SD.

### 2.20. Statistical Analysis

Data were analyzed using GraphPad Prism 6.0 Demo (GraphPad, San Diego, CA, USA). Statistical analyses of three or more groups of data were performed using ANOVA with Dunnett’s or Tukey’s multiple-comparisons test, as appropriate. A *t*-test was used to compare two groups of data. Values were expressed as their mean ± SD. Statistical significance was established at *p* < 0.05 (* *p* < 0.05, ** *p* < 0.01, *** *p* < 0.005 and **** *p* < 0.001), whereas ns was used to define no statistical significance (*p* > 0.05).

## 3. Results

### 3.1. Characterization of oEVs Loaded with SARS-CoV-2 mRNA

To evaluate oEV delivery of mRNA-based vaccine, oEVs were loaded with different mRNA of SARS-CoV-2, coding for: N protein (N), full S protein (FS) and the subunit 1 of S protein (S1) (see Table 1).

**Figure 1:**
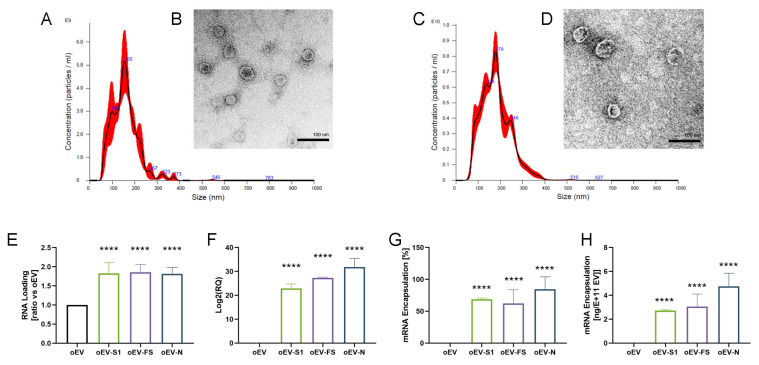
Delivery system characterization. (**A**–**D**) Representative images of oEVs and oEVs loaded with FS mRNA analyzed with nano-tracking analysis (NTA), and transmission electron microscopy (TEM). NTA of oEV (**A**) and oEV-FS (**C**) and TEM images of oEV (**B**) and oEV-FS (**D**). TEM scale bar 200 nm. (**E**) Total RNA was quantified in oEVs and oEVs loaded with S1, FS and N mRNAs (oEV-S1, oEV-FS, oEV-N) and expressed as ratio versus oEVs. (**F**) mRNA relative quantification was performed using qRT-PCR in oEVs and oEVs loaded with S1, FS and N mRNAs (oEV-S1, oEV-FS, oEV-N). (**G**) Percentage of mRNA encapsulated in oEVs following engineering with S1, FS and N mRNAs (oEV-S1, oEV-FS, oEV-N) in comparison to starting material. (**H**) Quantification of mRNA encapsulated into oEVs expressed as ng/10^11^ EV following engineering with S1, FS and N mRNAs (oEV-S1, oEV-FS, oEV-N). Data are represented as mean ± SD. **** *p* < 0.001.

Morphology and size analysis by NTA showed that oEVs loaded with mRNA had a biodistribution profile similar to unloaded oEVs. A small increase in size was observed after mRNA loading (Figure 1A,C), with a mean size of 167 ± 10 nm for empty oEVs and 227 ± 24 nm for loaded oEVs. TEM analysis demonstrated an intact membrane of oEV loaded with mRNA, with a round morphology and an electron-dense core (Figure 1B,D) similar to empty oEVs. RNA loading was similar for all three mRNA sequences, showing a comparable increase in RNA ratio with respect to control oEVs (Figure 1E). qRT-PCR analysis demonstrated a statistically significant mRNA loading of oEVs (Figure 1F) with a mean mRNA encapsulation efficiency of 72 ± 11% (Figure 1G) resulting in a loading capacity of 3.51 ± 1.09 ng/10^11^ oEVs (Figure 1H). Additional experiments with the mRNA coding for N protein showed that the co-incubation of oEVs and mRNA was less efficient than oEV loading (Figure S1A) and that the dose of mRNA loadable into oEVs could be increased up to 10 times in terms of total RNA (Figure S1B) and mRNA detected with qRT-PCR (Figure S1C).

Taken together, these data demonstrated that oEVs were efficiently loaded with different mRNA molecules.

### 3.2. mRNA Loaded into oEVs Was Resistant to Stress

**Figure 2:**
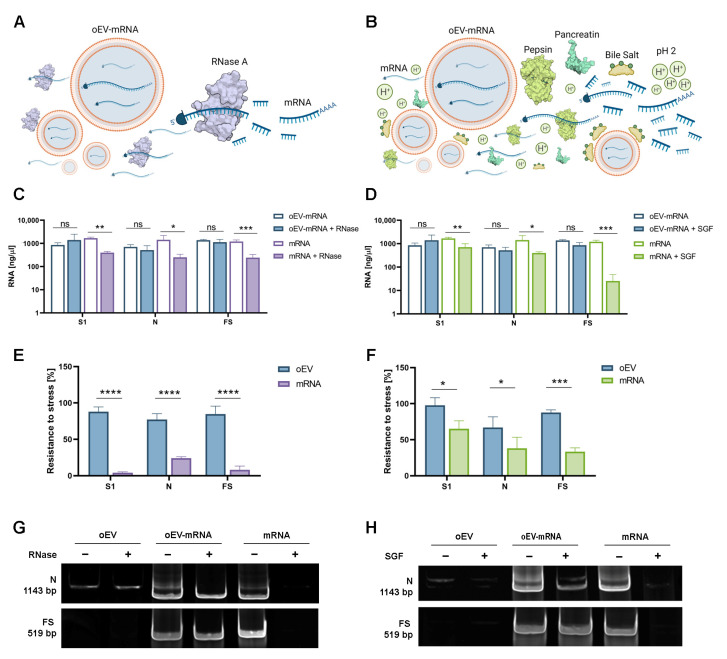
Evaluation of mRNA resistance to stress after loading into oEVs. Free mRNA (mRNA) or mRNA loaded into oEVs (oEV-mRNA) were subjected to two different degrading stresses: RNase A enzyme (**A**) and simulated gastric fluid (SGF) (**B**). The total RNA quantification (expressed as ng/µL) of S1, N, and FS mRNAs was measured when loaded into oEVs or not (free) and compared after treatment with RNase (**C**) or SGF (**D**). The specific resistance of each mRNA (S1, N, and FS) was quantified using qRT-PCR and expressed as the percentage of mRNA remaining after the treatment with RNase (**E**) or SGF (**F**). (**G**,**H**) Representative blots of PCR analysis for N and FS mRNAs in control oEVs (oEV), oEVs loaded with the mRNA (oEV-mRNA) and free mRNA before and after treatment with RNase (**G**) or SGF (**H**). Data are presented as mean ± SD. ns, not statistically significant. *p* > 0.05, * *p* < 0.05, ** *p* < 0.01, *** *p* < 0.005 and **** *p* < 0.001.

The ability of oEVs to protect mRNA from degradation due to RNase and to simulated gastric fluids (SGF) was evaluated in vitro. RNases are enzymes present in all environmental surfaces and are the main enzymes responsible for RNA degradation (Figure 2A), whereas gastric fluids represent the major stress in the gastrointestinal tract (Figure 2B). The integrity of mRNAs loaded into oEVs and free mRNAs was evaluated after RNase or SGF treatment. As shown in Figure 2C,D, respectively, the RNA amount did not change when mRNA was incorporated into oEVs, whereas free mRNA was significantly reduced after RNase and SGF treatment. Moreover, the analysis of each specific mRNA using qRT-PCR revealed that oEVs protected the mRNA in a statistically significant manner in comparison to free mRNA, with a mean percentage of resistance to stress of 83.3 ± 5.5% after RNase and 86.8 ± 28.0% after SGF for mRNA loaded into oEVs in comparison to 11.9 ± 10.5% after RNase and 54.8 ± 17.5% after SGF for free mRNA (Figure 2E,F). Additional experiments with N mRNA demonstrated that the co-incubation (without engineering) of oEV and mRNA did not protect mRNA from degradation (Figure S1D), confirming the poorer efficiency of the co-incubation technique in oEV loading. The protection was conferred by loading inside oEVs because lipid membrane permeabilization with Triton X-100 reduced the resistance to RNase (Figure S1E,F). These data were also confirmed by PCR experiments showing the disappearance of the mRNA-specific band after RNase (Figure 2G) and SGF (Figure 2H) of free mRNA but not of mRNA loaded into oEVs. Taken together, these data showed that the encapsulation of mRNA into oEVs makes them resistant to degrading conditions, which normally reduce free RNA integrity.

### 3.3. mRNA Was Delivered to Target Cells by oEVs and Translated into Protein

**Figure 3:**
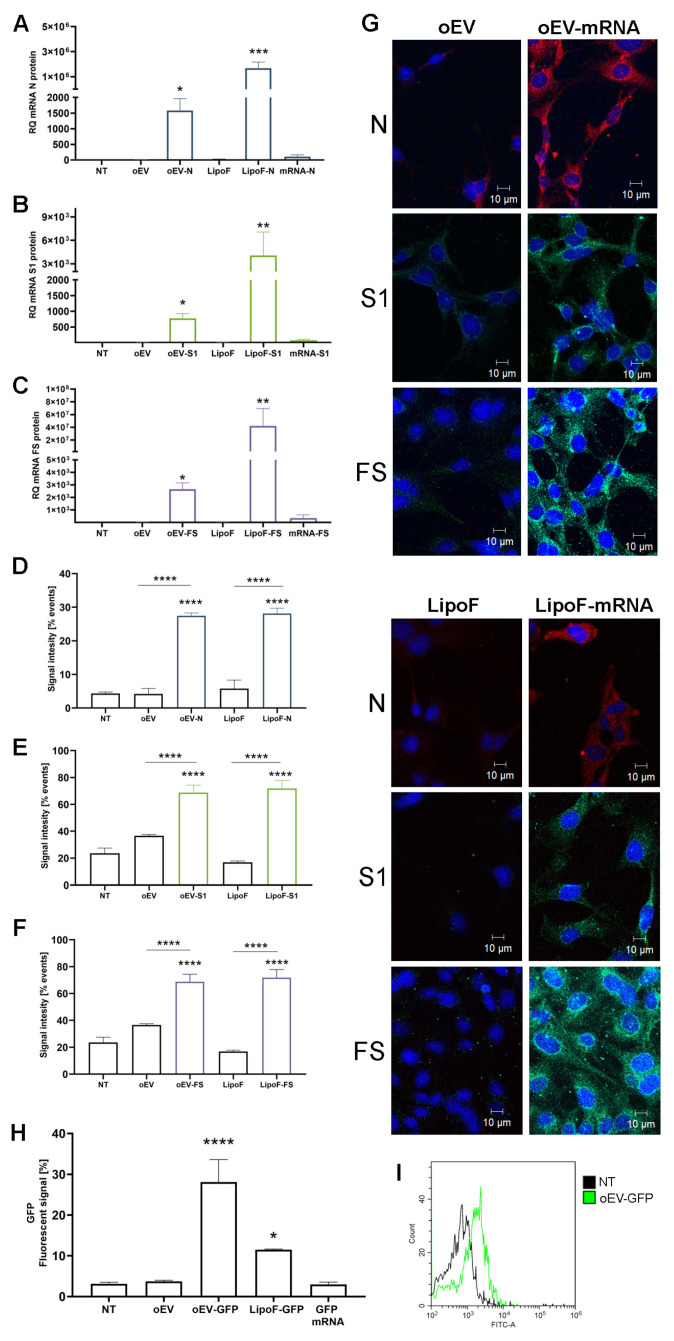
mRNA delivery to target cells and protein translation. Target cells were treated with oEVs loaded with N, S1 and FS mRNAs (oEV-N, oEV-S1, oEV-FS), and the mRNA uptake and translation into protein were evaluated after 24 h. (**A**–**C**) mRNA incorporation (expressed as RQ) was evaluated using qRT-PCR in comparison to untreated cells (NT) for N (**A**), S1 (**B**) and FS (**C**) mRNA. (**D**–**F**) The mRNA translation was measured by cytofluorimetric analysis for specific protein N (**D**), S1 (**E**) and FS (**F**) and compared to untreated cells (NT) or specific control oEV (for oEV-mRNA) and LipoF (for LipoF-mRNA). Different controls were used: untreated cells (NT), cells treated with oEVs (oEV), cells transfected with lipofectamine (LipoF) or lipofectamine with mRNA (LipoF-mRNA), and free mRNA. (**G**) Representative images of confocal microscopy of protein translation of N, S1 and FS in target cells after treatment with oEV loaded with mRNAs (oEV-mRNA) or transfected with lipofectamine and mRNA (LipoF-mRNA). As controls, unloaded oEVs (oEV) and transfection reagents (LipoF) were used. Photos are shown as merged images, with the nucleus stained with DAPI (blue) and N protein (red) or S1 and FS proteins (green). (**H**) Target cells were treated with GFP mRNA free (GFP mRNA), transfected with lipofectamine (LipoF-GFP) or loaded into oEVs (oEV-GFP), and fluorescent signal was measured by cytofluorimetric analysis and compared to untreated cells (NT). Treatment with unloaded oEVs (oEV) was used as an additional control. (**I**) Representative histogram showing cytofluorimetric measurement of GFP signal in untreated cells (NT, black line) and cells treated with oEVs loaded with GFP mRNA (oEV-GFP, green line). Data are presented as mean ± SD. * *p* < 0.05, ** *p* < 0.01, *** *p* < 0.005, **** *p* < 0.001.

The effective mRNA delivery to target cells and the translation into protein were evaluated. oEV treatment did not reduce target cells’ viability, as shown in Figure S2A. oEVs engineered with all three SARS-CoV-2 mRNA were incubated with macrophages used as APC cells. After 24 h, the delivery of mRNA was analyzed using qRT-PCR technique, showing the incorporation of each specific mRNA in APC. Classical transfection with lipofectamine was used as a control (Figure 3A–C). The mRNA translation into protein was detected by cytofluorimetric analysis (Figure 3D–F) and confirmed by confocal microscopy (Figure 3G), showing the expression of N, S1 and FS proteins in APC when mRNAs were transferred by oEVs or transfected with lipofectamine as the control. Finally, the ability of oEVs to transfer a functional mRNA was confirmed by APC expression of GFP protein 24 h after incorporation of GFP mRNA containing oEVs (Figure 3H,I). Moreover, the uptake of oEVs by target cells and the subsequent GFP expression were preserved after treatment with RNase and SGF (Figure S2B,C).

Together, these experiments demonstrated that oEVs delivered functional mRNA to target cells as it was translated into protein.

### 3.4. oEV-mRNA Activated Lymphocytes in an In Vitro Model

**Figure 4:**
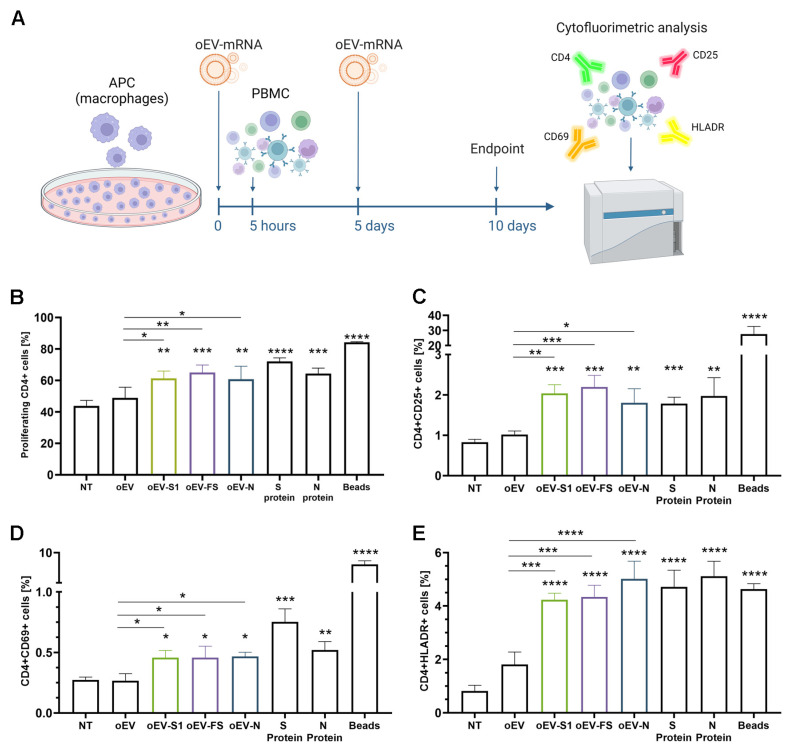
Evaluation of immune cell activation induced by oEV-mRNA using an in vitro model. (**A**) APC cells (macrophages) were treated with oEVs loaded with mRNA (oEV-mRNA) (S1, FS and N), co-incubated with PBMC after 5 h and re-stimulated after 5 days. At the experiment timepoint, cells were harvested and analyzed using a cytofluorimeter. As controls, cells were also untreated (NT), or treated with unloaded oEVs (oEV), S protein, N protein or T-Activator CD3/CD28 beads (Beads). (**B**) CD4+ proliferating cells were quantified as cells positive for CD4+ and CFSE staining. (**C**–**E**) Cells co-expressing CD4 and CD25 (**C**), CD69 (**D**) or HLADR (**E**) in all conditions are shown. Cell positivity is expressed as percentage of events detected. Statistical analysis was performed in comparison to NT treatment, and the oEV-mRNA effect was also compared to oEV treatment. Data are shown as mean ± SD. * *p* < 0.05, ** *p* < 0.01, *** *p* < 0.005, **** *p* < 0.001.

mRNA-based vaccines imply that the mRNA incorporated into APC is translated into protein and then presented to immune cells, triggering an immune response. To evaluate these steps in vitro, APC cells were co-incubated with oEV-S1, oEV-FS and oEV-N and, after 5 h, PBMC was added. Five days later, APCs were restimulated with the same treatments, and cells were analyzed at day 10 to detect lymphocyte activation, using cytofluorimetric analysis (see treatment scheme in Figure 4A). The treatment with oEVs loaded with all three mRNAs (oEV-S1, oEV-FS and oEV-N) induced a statistically significant activation of lymphocyte CD4+ in comparison to untreated cells (NT) and cells treated with control oEVs. In particular, cell activation was detected as an increase in cell proliferation by CFSE staining (Figure 4B), and a higher expression of activation markers CD25 (Figure 4C), CD69 (Figure 4D) and HLADR (Figure 4E). As positive controls, cells were stimulated with S and N proteins of SARS-CoV-2 and T-Activator CD3/CD28 beads. In conclusion, these data showed the ability of oEVs loaded with specific mRNA to activate an immune response in an in vitro model.

### 3.5. Mice Immunization via Different Administration Routes Induced Immune Response

**Figure 5:**
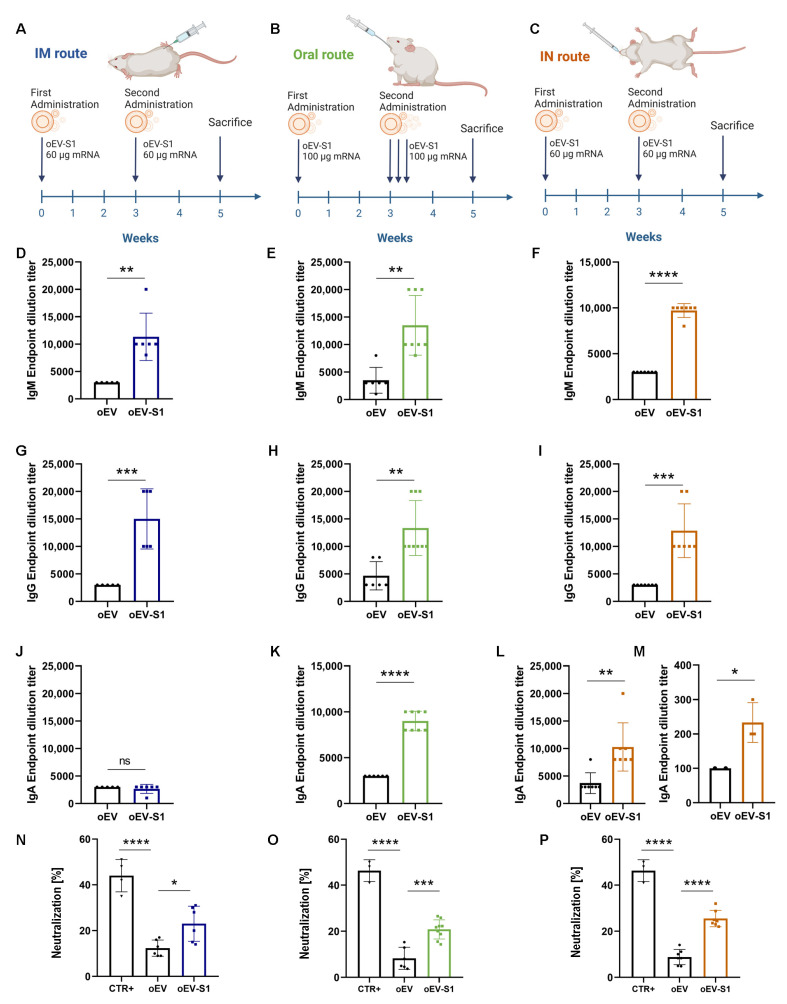
Mice vaccination and antibody detection. Mice were immunized with oEVs loaded with S1 mRNA (oEV-S1) or unloaded oEVs (oEV) via intramuscular (IM) (**A**), oral (**B**) and intranasal (IN) (**C**) routes. As shown, IM and IN immunization included a prime and a booster dose of 60 µg S1 mRNA, whereas oral immunization had a prime dose of 100 µg S1 mRNA and three booster doses in consecutive days. In all treatments, the two administrations were separated by three weeks and mice were sacrificed after five weeks from the first administration for subsequent analysis (see Material and Methods section). (**D**–**M**) S1-specific antibodies titer was measured by ELISA comparing serum of mice immunized with unloaded oEV or oEV-S1. IgM were analyzed for IM (**D**), oral (**E**) and IN (**F**) administration. IgG titer was determined in IM (**G**), oral (**H**) and IN (**I**) treatment. IgA antibodies were quantified with IM (**J**), oral (**K**) and IN (**L**) routes. (**M**) S1-specific IgA antibodies were also measured in bronchoalveolar lavage (BAL) fluid of IN administration. (**N**–**P**) Serum-derived neutralizing antibodies were also measured in IM (**N**), oral (**O**) and IN (**P**) administration. The percentage of neutralization induced by oEV-S1 or assay positive control (CTR+) was compared to unloaded oEVs. Data are presented as mean ± SD. ns, not statistically significant.* *p* < 0.05, ** *p* < 0.01, *** *p* < 0.005, **** *p* < 0.001.

To evaluate immunization in vivo, female balb/c mice were treated with oEV-S1 or empty oEV as the negative control, using intramuscular (IM), oral and intranasal (IN) administration as schematized in Figure 5A–C. IM and IN treatments were performed with a dose of 60 µg/mice and repeated after 3 weeks following the classical immunization scheme used for SARS-CoV-2 (Figure 5A,C) [29]. Since the gastrointestinal tract is highly dispersive for drugs, oral treatment was administered by gavage with a dose of 100 µg/mice, and the second administration was repeated for three consecutive days (Figure 5B). No treatments induced suffering in animals, with a weight increase similar to controls and the absence of histological alteration in tissue, suggesting a lack of toxicity (Figure S3). Moreover, the biodistribution following the oral and IN routes was assessed, confirming that oEVs targeted, respectively, the gastrointestinal and the respiratory tract (Figure S4). To assess the treatment immunogenicity, mice were sacrificed at the experiment timepoint and serum and splenocytes were collected to investigate immune stimulation.

Serum SARS-CoV-2-specific antibody titer demonstrated that all three administration routes elicited significant production of specific IgM (Figure 5D–F) and IgG antibodies (Figure 5G–I) in mice immunized with oEV-S1 with respect to mice treated with the unloaded oEVs used as the negative control. Of note, only oral and IN, but not IM, administration elicited a mucosal immune response with the formation of IgA antibodies (Figure 5J–L). For IN administration, the SARS-CoV-2-specific IgA antibody response was also measured in bronchoalveolar lavage (BAL) fluid, showing the presence of specific secretory IgA antibodies following immunization with oEV-S1 (Figure 5M). All antibody assays (IgM, IgG and IgA) were tested for specificity, and the IgG antibody assay was also analyzed for its sensitivity (Figure S5). Additional investigations revealed that serum samples contained neutralizing antibodies specific for SARS-CoV-2 following all three administration routes (Figure 5N–P).

**Figure 6:**
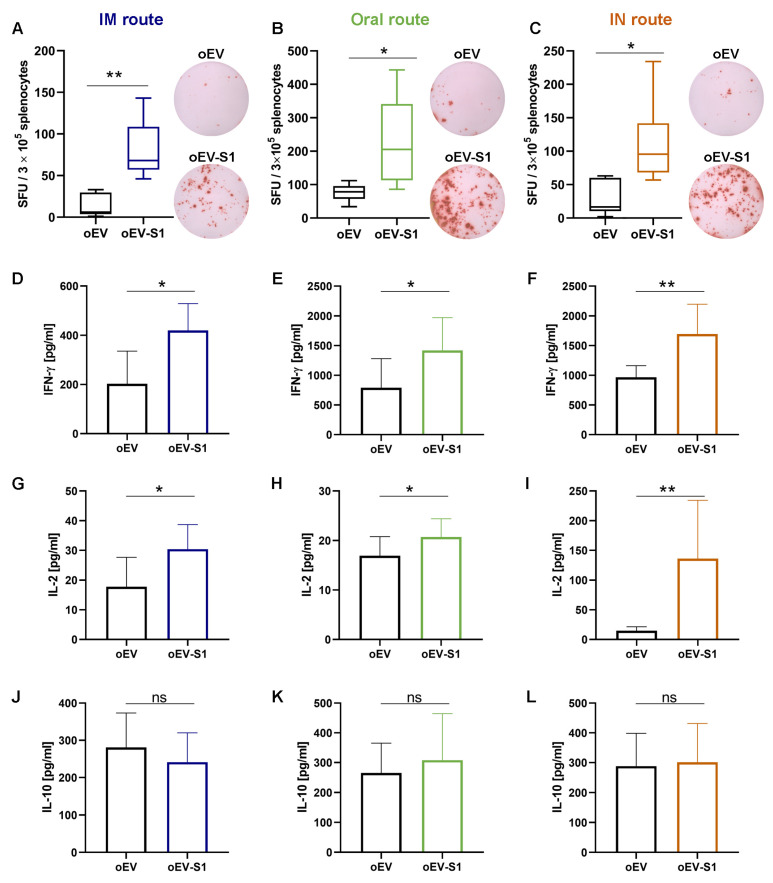
Cytokine secretion from splenocytes isolated from immunized mice via intramuscular (IM), oral and intranasal (IN) routes. (**A**–**C**) IFN-γ secretion by splenocytes was quantified upon stimulation with SARS-CoV-2 S peptide and expressed as spot-forming units (SFU) per 3 × 10^5^ cells following IM (**A**), oral (**B**) and IN (**C**). Representative spot images for each treatment were also recorded. (**D**–**L**) ELISA measurement of cytokine secretion by splenocytes after 24 h of stimulation with SARS-CoV-2 S peptide for IFN-γ for IM (**D**), oral (**E**) and IN (**F**) routes; IL-2 for IM (**G**), oral (**H**) and IN (**I**) routes; and IL-10 for IM (**J**), oral (**K**) and IN (**L**) routes, expressed as pg/mL. Statistical analysis was performed comparing splenocytes collected from mice immunized with unloaded oEVs (oEV) and oEVs loaded with S1 mRNA (oEV-S1). Data are expressed as mean ± SD. ns, not statistically significant. *p* > 0.05, * *p* < 0.05, ** *p* < 0.01.

Isolated splenocytes were stimulated with the SARS-CoV-2 S peptide ex vivo to investigate the cell-mediated immune response and verify the activation of Th1 (IFN-γ, IL-2) and Th2 (IL-10, -4). The immunization with oEV-S1 induced specific interferon γ (IFN-γ) secretion in comparison to the vehicle alone (oEV), detected using both ELISPOT assay (Figure 6A–C) and ELISA technique (Figure 6D–F). ELISA measurement of remaining cytokines demonstrated an increase in interleukin-2 (IL-2) in stimulated splenocytes collected by mice immunized with oEV-S1 with respect to oEVs (Figure 6G–I), whereas no enhancement of IL-10 was observed (Figure 6J–L) and IL-4 was not detected.

These findings indicate that vaccination with oEV-S1 using all three administration routes (IM, oral and IN) stimulated cell-mediated immune activation, which is associated with a Th1 cytokine (IL-2 and IFN-γ) rather than Th2 response (IL-10, -4).

**Figure 7:**
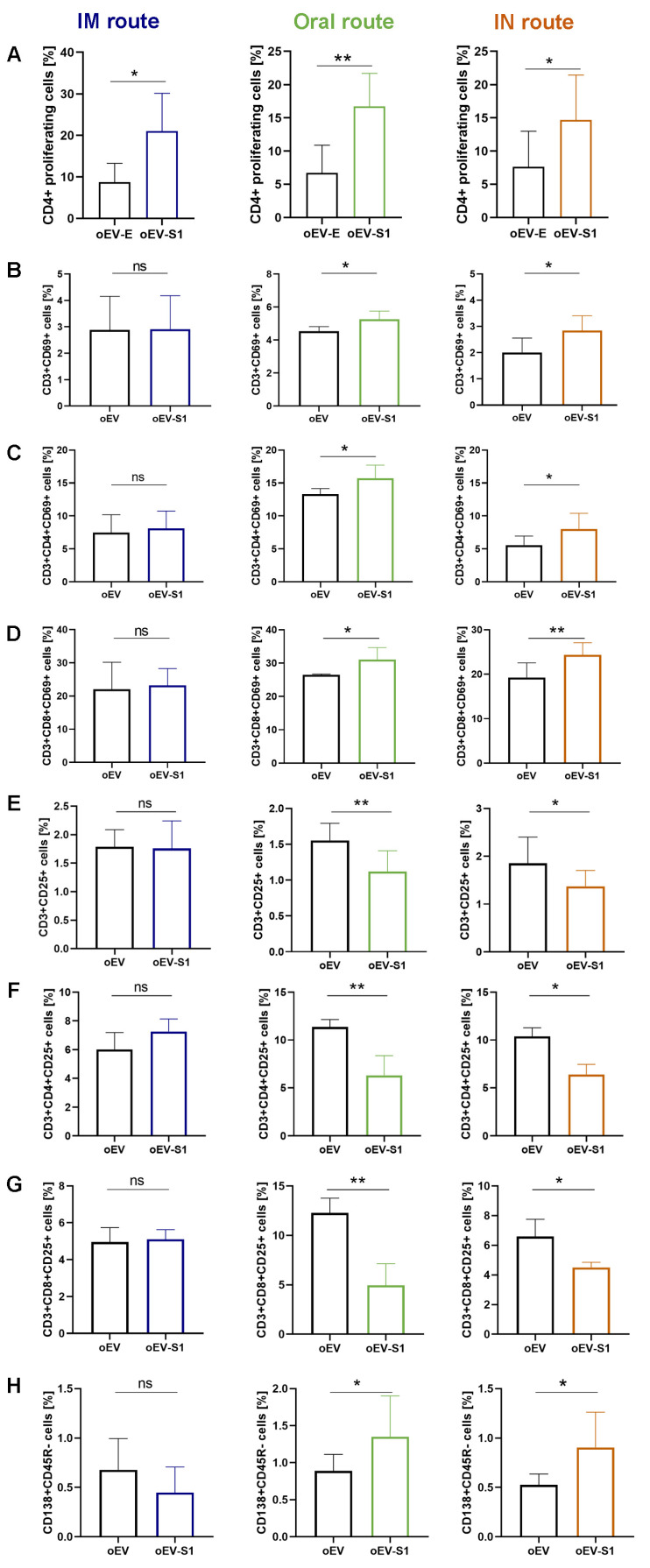
Immune cell activation following peptide stimulation ex vivo. Splenocytes isolated from immunized mice with unloaded oEVs (oEV) or oEVs loaded with S1 mRNA (oEV-S1) were stimulated with SARS-CoV-2 S peptide and their marker expression was analyzed by cytofluorimetric analysis for all three administration routes (IM, oral and IN). (**A**) CD4+ proliferating cells were quantified as cells positive for CD4+ and CFSE staining. Percentage of cells CD3+CD69+ (**B**), CD3+CD4+ co-expressing CD69+ (**C**), CD3+CD8+ co-expressing CD69+ (**D**), CD3+CD25+ (**E**), CD3+CD4+ co-expressing CD25+ (**F**), CD3+CD8+ co-expressing CD25+ (**G**) and CD138+CD45R− (**H**) are shown. Cell positivity is expressed as percentage of events detected. Statistical analysis compared splenocytes collected from mice immunized with unloaded oEVs (oEV) and oEVs loaded with S1 mRNA (oEV-S1). Data are expressed as mean ± SD. ns, not statistically significant. *p* > 0.05, * *p* < 0.05, ** *p* < 0.01.

Cytofluorimetric analysis of splenocytes upon stimulation with S peptide demonstrated a statistically significant increase in CD4+ lymphocytes following immunization with oEV-S1 in comparison to oEVs alone (Figure 7A), suggesting a specific immune cell activation. Additional investigations showed higher immune cell activation following oral and IN vaccination rather than IM administration. In fact, only mucosal routes (oral and IN), but not IM administration, were able to induce an increase in CD3+CD69+ (Figure 7B) with a higher presence of both CD3+CD4+ and CD3+CD8+ co-expressing CD69+ in mice treated with oEV-S1 in comparison to oEVs alone (Figure 7C,D). Moreover, only immunization with oral and IN routes, but not IM, significantly reduced the expression of regulatory T cell (T reg) CD3+CD25+ (Figure 7E) with a reduction in CD25+ expression in both CD3+CD4+ and CD3+CD8+ (Figure 7F,G). Notably, the presence of specific plasma cell CD138+CD45R− following treatment with oEV-S1 was also detectable after oral and IN but not after IM immunization (Figure 7H) [30]. In conclusion, these data demonstrated that all three administration routes activated a splenic immune cells response (CD4+) but only oral and intranasal immunization were able to induce a higher effect with an increase in specific plasma cells, an increase in CD69+ and reduction in CD25+ co-expression.

In order to also demonstrate in vivo the versatility of oEVs as mRNA carriers, a small number of animals were immunized with oEVs loaded with the bigger mRNA tested during in vitro coding for FS (oEV-FS) and compared to oEVs alone using all three administration routes (IM, oral, and IN). Meanwhile, oEVs efficiently delivered mRNA in vivo, inducing a humoral response with SARS-CoV-2 specific antibody and neutralizing antibodies with all three administration routes (Figure S6). Immune cell stimulation was also confirmed by the increased IFN-γ secretion from splenocytes stimulated with peptide ex vivo (Figure S7). Meanwhile, a change in IL-10 secretion was not observed with oEV-FS in comparison to oEV alone and IL-4 was undetectable, confirming the predominant activation of a Th1 rather than Th2 response (Figure S7). Finally, the splenic immune cell activation was confirmed using FS mRNA, and an increase in CD4+ cell proliferation was induced using all three administration routes (Figure S7).

Taken together, these data demonstrated that oEVs can efficiently deliver multiple SARS-CoV-2 mRNAs in vivo and immunize mice, inducing a specific humoral and cell-mediated immune response.

## 4. Discussion

In the present study, we provide a proof of concept that plant-derived EVs may represent a versatile platform for mRNA-based vaccines. Plant-derived EVs are an extractive natural product that may have several advantages with respect to synthetic nanoparticles [31]. The natural membrane envelope facilitates their uptake from target cells, they are not cytotoxic and they protect nucleic acids from enzyme degradation and environmental stress conditions [20,21,22,23,24,25]. Here, we used EVs purified from orange (*Citrus sinensis*) juice, which were expected to lack toxicity and immunogenicity due to their edible source and oral-induced tolerance. Being a natural product, plant EVs can be purified using a simple extraction process that does not require expensive cell culture conditions, and their ability to transfer a great number of biomolecules makes them a good candidate for drug delivery [19,20,21,22,23,24]. As a prototype of an mRNA-based vaccine, to evaluate the efficacy of oEVs as a carrier, we selected mRNA coding for SARS-CoV-2 antigens.

In the present study, we demonstrated that oEVs can be efficiently loaded with different mRNA molecules with sizes ranging from 670 to 3820 nt, with a similar encapsulation efficiency and without altering oEVs’ integrity. Previous studies on mammalian EVs have shown that membrane vesicles confer stability to nucleic acid from degradation and from low and high pH conditions [32,33,34]. Here, we demonstrated that mRNA carried by engineered oEVs was highly protected from degradation induced by treatment with RNase enzyme and simulated gastric fluid (SGF) in vitro. Importantly, mRNA conveyed by oEVs was efficiently delivered to target cells and translated into protein. In the target cell, the protein was functional and was able to be presented by APC cells and stimulate a lymphocyte response, detected as the increase in CD4+ proliferating cells and of CD69+, CD25+ and HLADR+ activated cells (Figure 4) [35,36]. These findings suggested the possible efficacy of oEVs as a carrier for mRNA-based vaccines and prompted us to test their effect in mice.

In the vaccine field, the interest in EV-mediated antigen delivery is growing based on the observation that mammalian cells infected with viral pathogens release EVs containing viral constituents able to trigger an immune response [37]. Moreover, EVs carrying S protein have been detected in infected patients, and their potential role in inducing a humoral specific immune response has been suggested [38,39]. Interestingly, compared to soluble antigens, EV-associated antigens were shown to elicit a stronger cytotoxic CD8+ T response [18]. Mammalian cells engineered to express a S and N protein in EVs were shown to efficiently immunize mice after IM injections [16].

Here, we selected the well-studied mRNA coding for subunit S1 of SARS-CoV-2 and demonstrated that IM injection in mice elicited a humoral immune response with the production of specific IgM and IgG against S1 protein, the induction of neutralizing antibodies and a T cell immune response. Splenocytes stimulated with the S peptide exhibited a significant increase in CD4+ proliferating cells upon immunization with oEV-S1 with respect to oEV alone, suggesting that the oEV formulation enables general CD4+ cell activation. The same effect on the increase in CD4+ was also observed in other studies, for instance, Perex et al. [39] when using IN administration of a modified vaccinia virus Ankara (MVA)-based vaccine, and Corbett et al. [29] via IM immunization with mRNA-1273.

In this study, immunization with oEV-S1 stimulated a cell-mediated immune response associated with a Th1 cytokine (IFN-γ, IL-2) rather than a Th2 response (IL-10, IL-4). In fact, we observed an increase in the secretion of IFN-γ and IL-2 from splenocytes stimulated with the S peptide, whereas no difference was detected for IL-10 and IL-4. These data are in accordance with other studies demonstrating the major activation of a Th1 response following vaccination with mRNA coding for the SARS-CoV-2 antigen. Corbett et al. [29] reported that mice vaccinated with mRNA-1273 elicited a predominant Th1 response, detected as production of cytokines IFN-γ, TNF and IL-2 by total CD4 T cells under ex vivo stimulation with SARS-CoV-2 peptide pools. In a study by Zhang et al. [39], the authors showed that splenocytes isolated from mice immunized with SARS-CoV-2 RBD and in vitro re-stimulated with peptide pools covering the SARS-CoV-2 RBD induced a Th1-biased response with a significant increase in IFN-γ and IL-2 secretion, but no differences for Th2 response (IL-6, IL-4) were observed [40]. Moreover, cytokine polarization analysis performed by Sahin et al. [41] on PBMC collected by patients vaccinated with BNT162b1 showed secretion of IFN-γ and IL-2 but most individuals failed to secrete IL-4 [41]. IL-10, instead, was associated with Th2 response in SARS patients [42] and with Th2 response upon vaccination [43].

Since more than 90% of the immune system is located in the gastroenteric tract, we also vaccinated mice via the oral route. Oral immunization induced an effective humoral and cellular immune response, similar to IM administration, indicating efficient protection of mRNA carried by oEVs from gastric degradation in vivo. Indeed, plant EVs are good candidates for oral drug delivery due to their resistance to acidic and basic conditions and to efficient intestinal absorption [7,44,45].

Besides the oral route, another suitable site of administration is represented by the intranasal tract with the presence of many immune cell components able to trigger an immune response, such as macrophages and T- and B-lymphocytes [46]. Here, we observed that an intranasal administration of oEV formulation with S1 mRNA was able to trigger a humoral and cellular immune response similar to oral immunization. The intranasal route for EV-based vaccine was confirmed by a previous report using Salmonella typhimurium EV decorated with the Spike receptor-binding domain derived from mammalian cell culture. In this model, intranasal immunization of the golden Syrian hamster resulted in a high level of serum anti-Spike receptor-binding domain IgG as well as a mucosal response [17]. Wang et al. [47] showed that human lung-derived EVs conjugated with recombinant SARS-CoV-2 receptor-binding domain efficiently immunized mice and reduced inflammation after a challenge with live SARS-CoV-2 in hamsters.

Of interest, both the oral and the intranasal vaccination not only stimulated the specific IgM and IgG antibody production but also the induction of a specific secretory IgA response, a critical component in the mucosal first barrier adaptive immune response [48]. This finding is consistent with the observation that strategies based on mucosal vaccination stimulate IgA secretion in the mucosa [49]. Hassan and colleagues previously showed that an intranasal dose of a chimpanzee adenovirus-vectored vaccine encoding a prefusion stabilized spike protein (ChAd-SARS-CoV-2-S) promoted systemic and mucosal IgA in mice and macaques [50,51,52]. The study of Popowski et al. [53] reported the vaccination by direct inhalation using nebulization of lyophilized EVs derived from lung or from conditioned medium of HEK 293T cell line previously engineered with the S protein mRNA via electroporation. When compared with liposomes, mRNA-loaded EVs elicited significantly higher production of specific IgG and secretory IgA antibodies, suggesting that EVs are superior to synthetic liposomes as carriers of mRNA-based vaccines [53]. As expected, the biodistribution of oEVs revealed different accumulations when using the intranasal or oral route, showing efficient mRNA delivery and mice immunization in both administration protocols.

Although this did not apply to the intramuscular route, oral and intranasal immunization were shown to also induce increased cell-mediated immune activation. These data are consistent with a previous observation that mucosal vaccine administration can provide not only local but also systemic protection [49,54]. In this study, marker expression of peptide-stimulated splenocytes revealed that oral and IN immunization with oEV-S1 induced an increase in the lymphocyte activation marker CD69+ in comparison with oEV vaccination. We observed an increase in CD69+ expression on both total CD3+ and CD3+CD4+ and CD3+CD8+. This finding was supported by previous studies that reported the induction of CD8+CD69+ [55] and CD4+CD69+ [56] following vaccination against SARS-CoV-2 [57]. Here, oEV-S1 treatment via the oral and IN routes induced a significant reduction in CD3+, CD3+CD4+, and CD3+CD8+ co-expressing CD25+, a typical marker of regulatory T cell (T reg). CD25 (or the IL-2 receptor α-chain) is considered a key regulator of immune response since its high affinity to IL-2 induces IL-2 deprivation, promoting an important immunosuppressive activity that prevents harmful effects due to immune system hyperstimulation [58]. The inhibitory effects of Tregs on immune response induced post-vaccination have been experimentally demonstrated in different mice models of Treg depletion [58]. T reg depletion was proven to increase vaccine-derived protection in an influenza virus infection model [59] and induced a more durable antibody response to mRNA-COVID-19 vaccination in patients with plasma cell dyscrasia [60]. In this study, mice immunized with oEV-S1 via mucosal routes (oral and IN) also exhibited an increased number of splenic plasma cells in comparison to mice treated with oEV vehicle alone. A similar result was also reported by Gao et al. [61] as an increase in the ratio of CD19−CD138+ plasma cells in total lymphocytes derived from the spleen of mice immunized with RBD9.1 peptide.

Finally, the versatility of oEVs to deliver different immunizing mRNAs was demonstrated using oEVs loaded with FS mRNA in a small number of animals, showing a similar immune response to vaccination with oEV-S1 using all three administration routes (IM, oral, and IN).

## 5. Conclusions

In conclusion, we successfully demonstrated that EVs derived from edible plants can serve as a carrier for the therapeutic exploitation of nucleic acids. In fact, they confer protection from degradation and allow for efficient delivery to mammalian cells in vitro and in vivo. They may be suitable for the delivery not only of mRNA but also of other nucleic acid molecules, such as siRNA, miRNA, and DNA. In the present study, we demonstrated that mRNA from a viral protein can be loaded in orange-derived EVs, eliciting an immune response with antibody production and immune cell activation. We tested SARS-CoV-2 mRNAs as a proof of concept, but the cargo can be adjusted for targeting different pathogens, paving the way to several applications in the vaccine field. Among administration routes, oral and intranasal represent several advantages as needle-free techniques and were demonstrated to be superior to the IM route, also conferring IgA mucosal protection and increased splenic cell activation. Therefore, edible plant EVs may represent a platform for the delivery of different therapeutic molecules, especially for mucosal absorption.

## Figures and Tables

**Table 1:** Selected mRNA sequences from SARS-CoV-2, genomic sequence NC_045512.2.

mRNA Name (Abbreviation)	Gene Symbol (NCBI)	Gene Description	ORF	Nucleotide Position	Nucleotide Number	Reference
N	N	nucleocapsid phosphoprotein	ORF 9	28274–29533	1260 nt	GENE, FASTA
FS	S	surface glycoprotein	ORF 2	21563–25384	3822 nt	GENE, FASTA
S1	S1	surface glycoprotein, Spike-S1-RBD	ORF 2	22518–23186	669 nt	GENE, FASTA

## Data Availability

Data are contained within the article.

## Supplementary Materials

The following supporting information can be downloaded at: https://www.mdpi.com/article/10.3390/pharmaceutics15030974/s1, Figure S1: oEV loading with an increasing amount of mRNA; Figure S2: Cytotoxicity and uptake of oEV and oEV-GFP in target cells; Figure S3: Mice weight during vaccine treatment via multiple administration routes; Figure S4: Biodistribution of oral and IN administration in vivo; Figure S5: ELISA specificity and sensibility for antibody titer measurement; Figure S6: Antibody production following mice immunization with oEV-FS; Figure S7: Splenic cell activation upon vaccination with oEV-FS; Table S1: Primer sequences used for qRT-PCR and PCR experiments.
