# Supplementary material for: Plant-Derived Extracellular Vesicles as a Delivery Platform for RNA-Based Vaccine: Feasibility Study of an Oral and Intranasal SARS-CoV-2 Vaccine
Source: Pharmaceutics. 2023 Mar 17;15(3):974. doi: 10.3390/pharmaceutics15030974 (PMC10058531; doi:10.3390/pharmaceutics15030974)
Supplement: Supplementary file 1 [file pharmaceutics-15-00974-s001.zip › pharmaceutics-2251281-supplementary.pdf]

Supplementary information

Plant-Derived Extracellular Vesicles as a Delivery Platform for RNA-Based Vaccine: Feasibility Study of an Oral and Intranasal SARS-CoV-2 Vaccine

Margherita A. C. Pomatto [1,2,*], Chiara Gai [1,2], Federica Negro [1], Lucia Massari [1], Maria Chiara Deregibus [2], Cristina Grange [2], Francesco Giuseppe De Rosa [2] and Giovanni Camussi [1,2,*]

[1] EvoBiotech s.r.l., 10122 Turin, Italy
[2] Department of Medical Science, University of Turin, A.O.U. Città della Salute e della Scienza di Torino, 10126 Turin, Italy
* Correspondence: mpomatto@evobiotech.it (M.A.C.P.); giovanni.camussi@unito.it (G.C.)

Figure S1

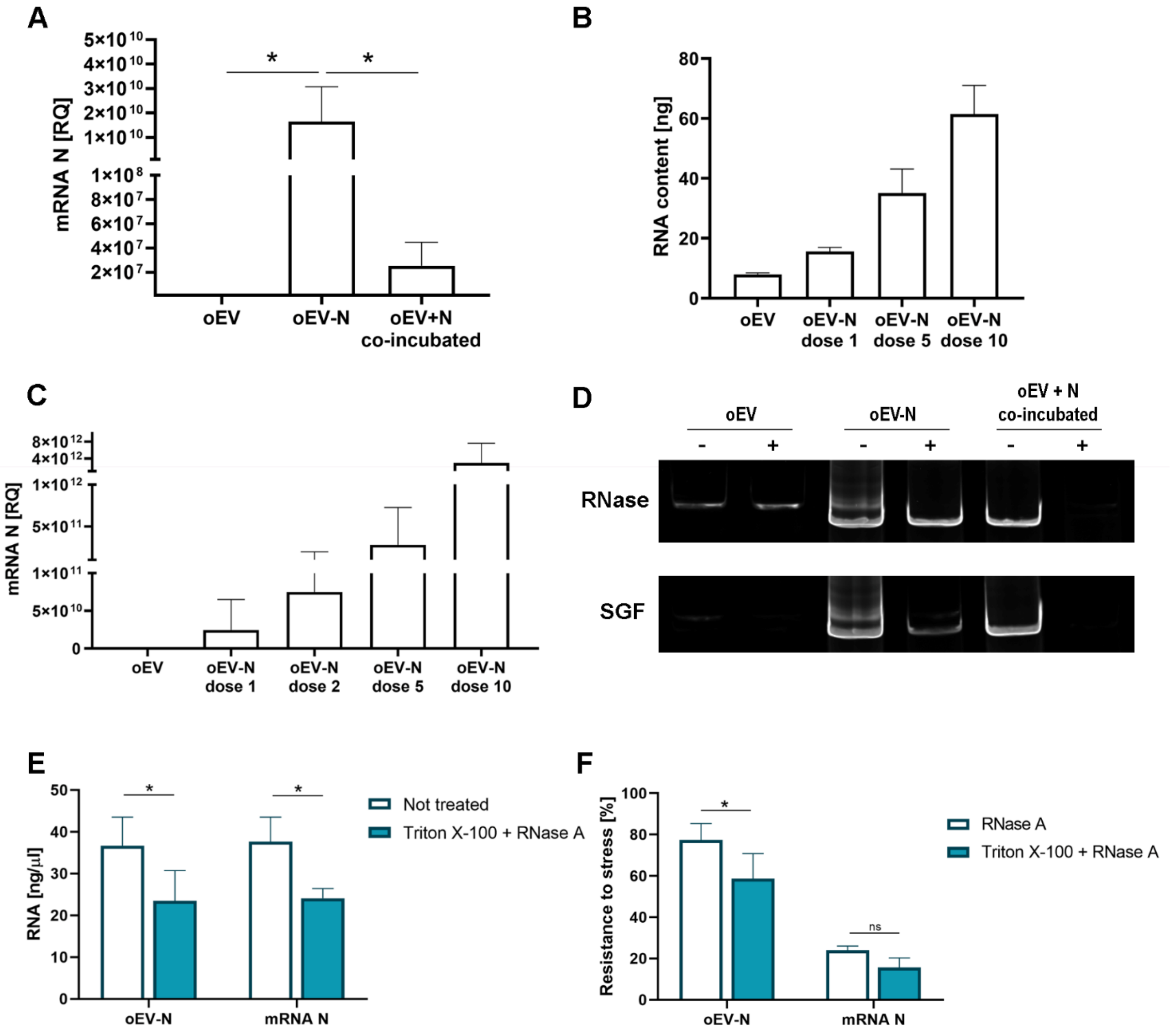

oEV loading with N mRNA. (A) oEV loading was analyzed by qRT-PCR and expressed as RQ to evaluate the N mRNA presence in control unloaded oEVs (oEV), after loading with oEVs (oEV-N) or after co-incubation of oEVs with mRNA without engineering (oEV+N co-incubation). (B-C) oEVs were loaded with increasing doses of N mRNA (oEV-N) starting from the mRNA dose used for experiments shown in a (dose 1= 0,42 µg/10^{10} EV, dose x2, x5, and x10). (B) Total RNA content expressed as total ng, (C) mRNA expression (RQ) measured by qRT-PCR. (D) Representative images of the comparison of loading and co-incubation evaluated by PCR experiment before and after treatment with RNase or Simulated Gastric Fluid (SGF). (E-F) oEVs were treated with Triton X-100 to permeabilize their lipid membrane before undergoing RNase treatment and molecular analysis. (E) Total RNA content expressed as concentration ng/µl and comparison between not treated samples and samples treated with Triton X-100 and RNase, (F) Percentage of mRNA resistance after treatment measured by qRT-PCR and comparison of samples treated with only RNAse A or combination of Triton X-100 and RNAse A. Data are presented as mean ± SD. ns (not statistically significant) >0.05, *$p < 0.05$.

Figure S2

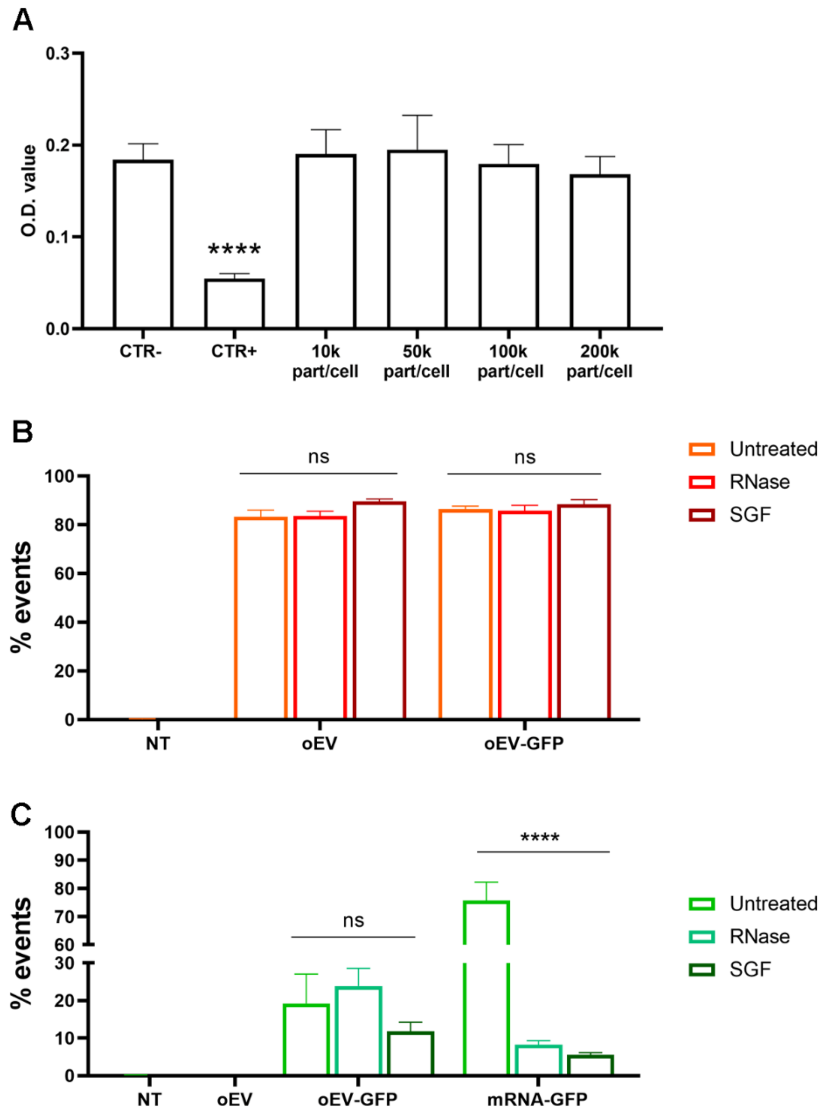

Cytotoxicyty and uptake of oEVs and oEV-GFP in target cells. (A) Target cells were treated with increasing dose of oEVs and cell viability was determined by MTT assay after 24 hours of treatment with: culture medium (NT), culture medium added with 50% DMSO (CTR+), different doses of oEVs (10k, 10,000 particle/cell; 50k, 50,000 particle/cell; 100k, 100,000 particle/cell; 200k, 200,000 particle/cell). Statistical analysis compared NT with other treatments. (B) The uptake of oEVs stained with a fluorescent dye (PKH26) was measured as percentage of positive events by cytofluorimetric analysis. Target cells were treated with medium (NT) or medium plus unloaded oEVs (oEV) or oEVs loaded with GFP mRNA (oEV-GFP) subjected or not (untreated) to RNase treatment (RNase) or simulated gastric fluid degradation (SGF). After 24 hours, the fluorescent signal was compared between doses untreated or treated with RNase or SGF. (C) The GFP signal resulting from mRNA translation was also measured. As an additional control, cells were directly transfected with mRNA, and signal reduction was evaluated after treatment with RNase or SGF. Data are presented as mean ± SD. ns (not statistically significant)> 0.05, and ****p < 0.001.

Figure S3

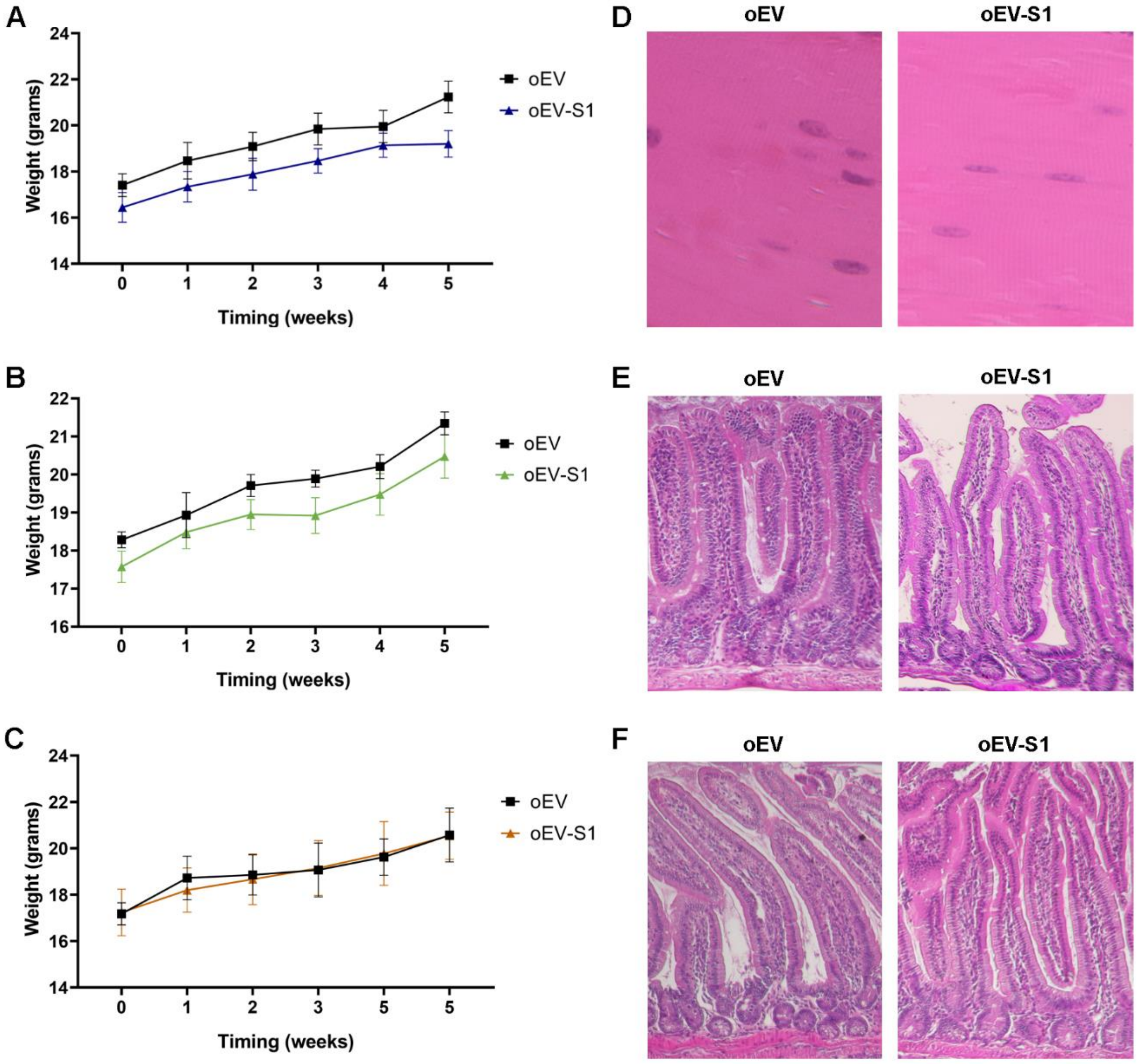

Mice weight during vaccine treatment *via* multiple administration routes and hystological analysis. (A-C) Mice weight (expressed in grams) was monitored during all experiment from first administration to sacrifice for intramuscular (IM) (A), oral (B), and intranasal (IN) (C) administration. Data are expressed as mean ± SD. (D-F) H&E hystological representative images of mice treated with control unloaded oEVs (oEV) or oEVs loaded with S1 mRNA (oEV-S1) of leg muscle after intramuscular injection (20X magnification) (D) or of the intestine (10X magnification) after oral (E) and intranasal (F) administration.

Figure S4

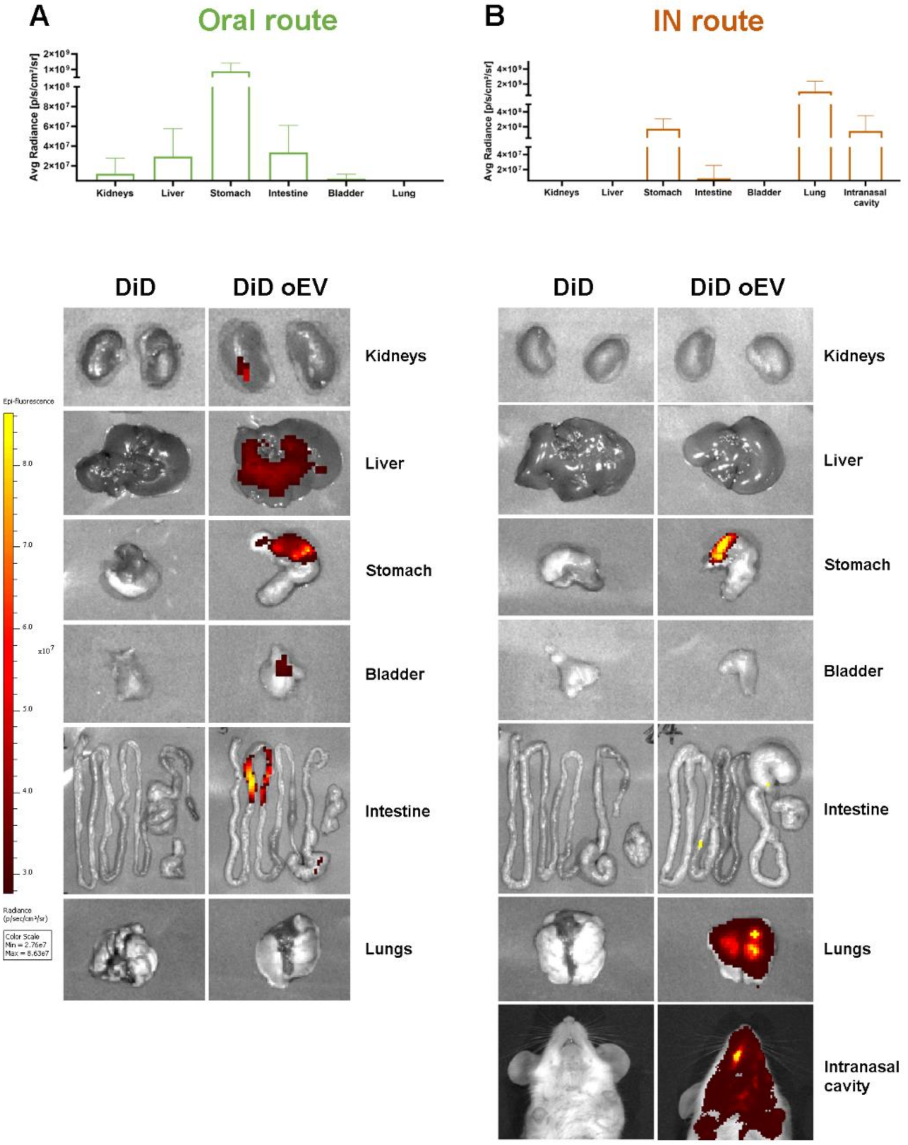

Biodistribution of oral and IN administration *in vivo*. Biodistribution of oEVs administered in mice using oral (A) and intranasal (IN) (B) administration was evaluated. For each route, the biodistribution in organs was showed in the graph expressed as averange radiance (p/sec/cm^2/sr), and representative images for each organ were indicated. oEVs were stained with DiD dye and the same amount od DiD dye used to label oEVs was administered to subtract the signal background. Data are presented as mean ± SD.

Figure S5

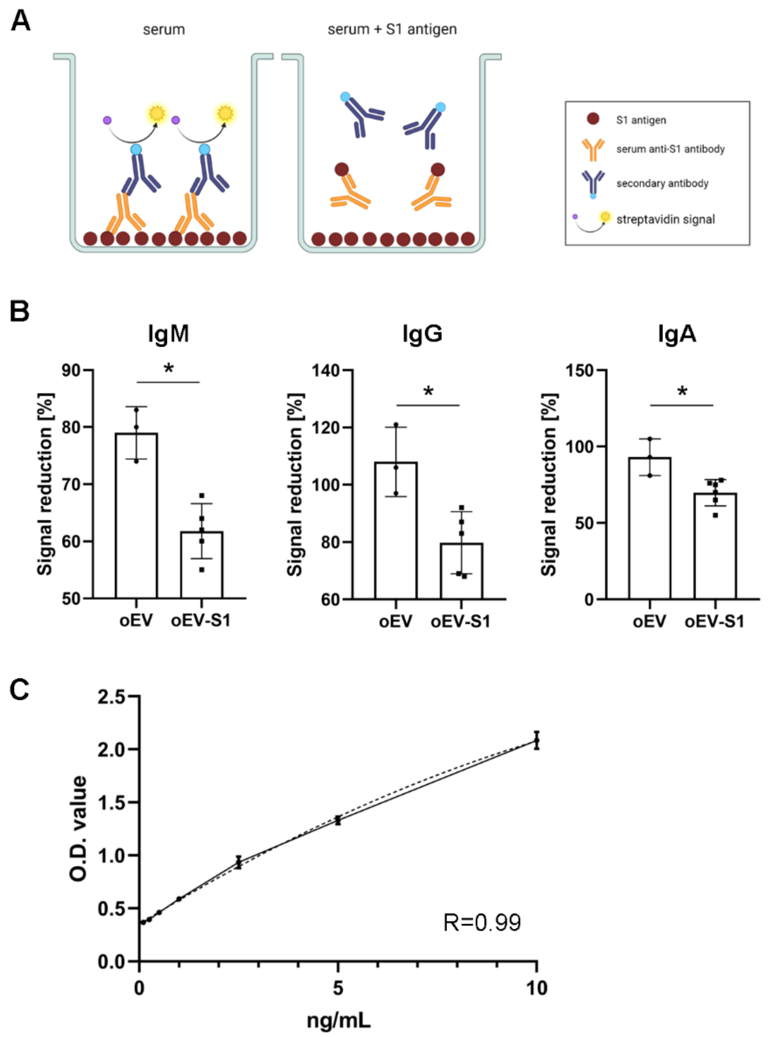

ELISA specificity and sensibility for antibody titer measurement. (A) ELISA specificity was determined using a competition assay. Antigen specificity was measured as reduction of specific signal due to previous co-incubation with an excess of S1 protein, as shown in scheme. (B) Signal reduction was compared in serum derived by mice immunized with unloaded oEVs (oEV) or oEVs loaded with S1 mRNA (oEV-S1) for IgM, IgG, and IgA. (C) Assay sensibility was tested for IgG antibodies directed against SARS-CoV-2 S1 protein showing O.D. values and protein concentration (ng/mL) and the linear curve R^2. Data are presented as mean ± SD. $^*p < 0.05$.

Figure S6

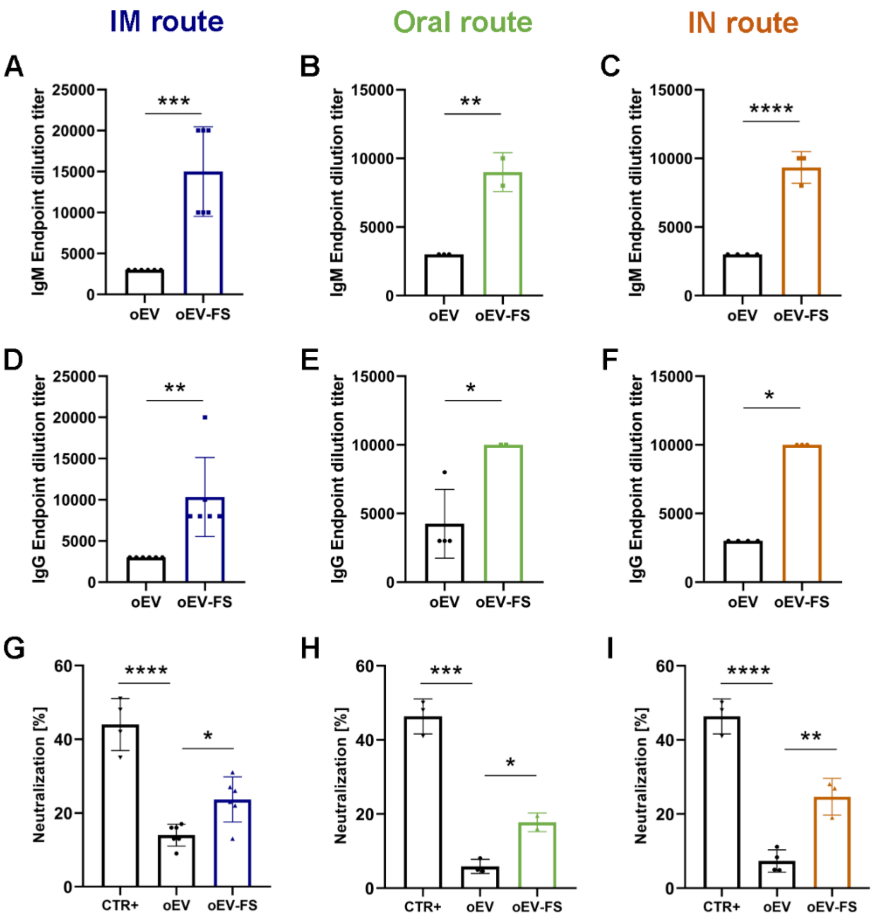

Antibody production following mice immunization with oEV-FS. (A-F) Antibody titer was measured by ELISA comparing serum of mice immunized with with oEVs loaded with FS mRNA (oEV-FS) or unloaded oEVs (oEV). The titer of S1-specific IgM titer was determined following intramuscular (IM) (A), oral (B) and intranasal (IN) (C) administration. The titer of S1-specific IgG titer was determined following intramuscular (IM) (D), oral (E), and intranasal (IN) (F) administration. (G-I) The neutralization capacity of serum-derived antibodies was tested as a percentage of neutralization induced by oEV-FS or assay positive control (CTR+) compared to unloaded oEV in IM (G), oral (H), IN (I) route. Data are presented as mean ± SD. *$p < 0.05$, **$p < 0.01$, ***$p < 0.005$, and ****$p < 0.001$.

Figure S7

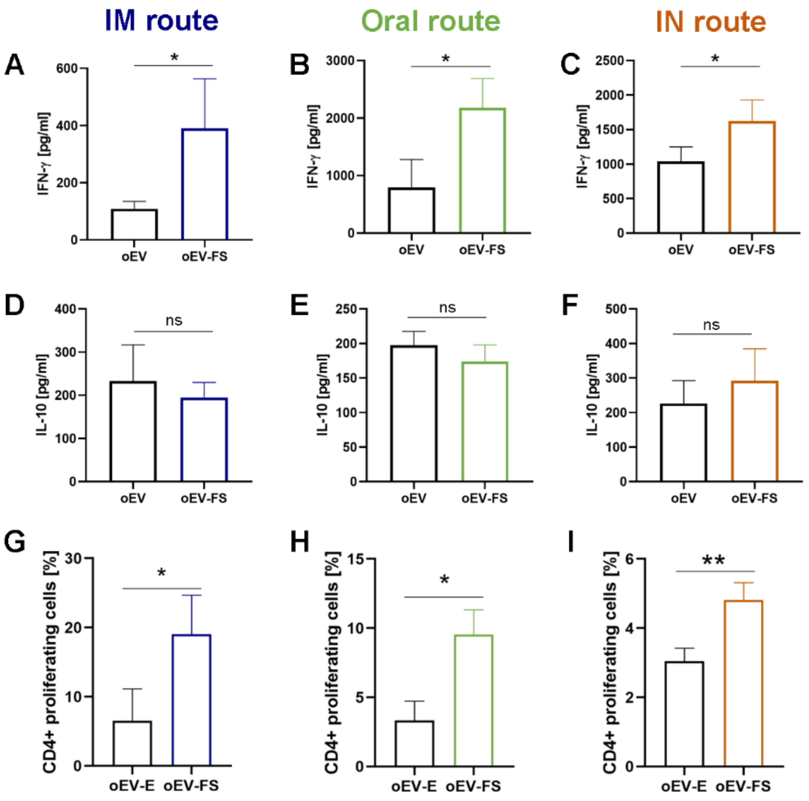

Splenic cell activation upon vaccination with oEV-FS. (A-F) ELISA measurement of cytokine secretion by splenocytes isolated by immunized mice (via IM, oral and IN route) after 24 hours of stimulation with SARS-CoV-2 S peptide for IFN-γ (A-C) and IL-10 (D-F) expressed as pg/mL. (G-I) Citofluorimetric analysis quantified CD4+ proliferating cells as percentage of cells (event %) positive for CD4+ and CFSE staining after five days of co-incubation with SARS-CoV-2 S peptide. Statistical analysis was performed comparing splenocytes collected from mice immunized with unloaded oEVs (oEV) and oEV loaded with S1 mRNA (oEV-S1). Data are expressed as mean ± SD. ns (not statistically significant) > 0.05, *p < 0.05, and **p < 0.01.

Table S1

Primer sequences used for qRT-PCR and PCR experiments.

Name	Forward Primer (F)	Reverse Primer (R)	Application
ACTB	CATGTACGTTGCTATCCAGGC	CTCCTTAATGTCACGCACGAT	qRT-PCR
cel-mir-39	CACCGGGTGTAAATCAGCTTG	-	qRT-PCR
N	ACCCGCAATCCTGCTAACAA	CAAGCAGCAGCAAAGCAAGA	qRT-PCR
S1	GCCGGTAGCACACCTTGTAA	ACACCTGTGCCTGTTAAACCA	qRT-PCR
N	TGGACCCCAAAATCAGCGAA	CTCTGCGGTAAGGCTTGAGT	PCR
FS	ACTGTGCACTTGACCCTCTC	CATGAGGTGCTGACTGAGGG	PCR
